# Improved Estimates of Biomass Expansion Factors and Root‐To‐Shoot Ratios: An Approach for Different Forest Types Across a Climatic Gradient in Brazil

**DOI:** 10.1111/gcb.70395

**Published:** 2025-08-04

**Authors:** Isáira Leite e Lopes, Otávio Camargo Campoe, Geovanni Malatesta Barros, Anny Francielly Ataide Gonçalves, Gabriela Gonçalves Moreira Matzner, Marco Aurelio Figura, Clayton Alcarde Alvares, Fernanda Leite Cunha, Verônica Boarini Sampaio de Rezende, James Stahl, Josiana Jussara Nazaré Basílio, Hyngrid Jaiely Araújo Félix, Thiago Bortoluzzi Boigues, Grasiele Dick, Eduardo Moré de Mattos, Joannès Guillemot, Guerric Le Maire, Rachel Cook, Timothy J. Albaugh, Rafael Alejandro Rubilar, Márcia Silva de Jesus, Adriano Scarpa Tonaco, Juliana Soares Biruel Munhoz, Isabel Deliberari, Jose Luiz Stape, Yann Nouvellon, Jean‐Paul Laclau

**Affiliations:** ^1^ Department of Forest Sciences Federal University of Lavras – UFLA Lavras Brazil; ^2^ Department of Forestry and Environmental Resources North Carolina State University – NCSU Raleigh North Carolina USA; ^3^ Department of Forest, Soils, and Environmental Sciences São Paulo State University “Júlio de Mesquita Filho” – UNESP Botucatu Brazil; ^4^ Forest Research and Development (R&D) of Bracell Lençóis Paulistas Brazil; ^5^ Forest Research and Development (R&D) of Klabin SA Telêmaco Borba Brazil; ^6^ Forest Research and Development (R&D) of Suzano SA Limeira Brazil; ^7^ Institute of Forestry Research and Studies – IPEF Piracicaba Brazil; ^8^ Forest Research and Development (R&D) of CMPC Guaíba Brazil; ^9^ Geplant Forest Technology Ltd. Piracicaba Brazil; ^10^ CIRAD UMR Eco&Sols Montpellier France; ^11^ Eco&Sols University of Montpellier, CIRAD, INRAe, Institut Agro, IRD Montpellier France; ^12^ Department of Forest Sciences Luiz de Queiroz College of Agriculture, University of São Paulo – USP/ESALQ Piracicaba Brazil; ^13^ Department of Forest Resources and Environmental Conservation Virginia Tech Blacksburg Virginia USA; ^14^ Forestry Department University of Concepción – UDEC Concepción Chile; ^15^ Brazilian Tree Industry – Ibá São Paulo Brazil

**Keywords:** allometry, carbon, climate variables, forest sector, greenhouse gas inventory, modeling

## Abstract

Advancements in the current state of the art of the key drivers of biomass expansion factor (BEF) and the root‐to‐shoot ratio (*R*) are crucial for producing accurate information on forest biomass and carbon stocks. Hence, we compiled a nationally representative dataset encompassing diverse tree growth stages and climatic gradients. In this study, we propose models to improve BEF and *R* estimates at the tree level for *Eucalyptus* and *Pinus* plantations in Brazil. In general, the BEF values were more representative (91.7%) in the database than the *R* values (8.3%) due to the high cost of collecting coarse roots. Regarding genera, *Eucalyptus* was more extensively sampled (89.9%) than *Pinus* (10.1%), reflecting the predominance of *Eucalyptus* as the most widely planted genus in Brazil. The average BEF and *R* values calculated in this study were 1.16 and 0.22, respectively, for *Eucalyptus* spp. and 1.22 and 0.31, respectively, for *Pinus* spp. In predicting the BEFs, the random effects in the linear mixed model that significantly captured the variations in *Eucalyptus* and *Pinus* spp. were temperature and age class, respectively. The fixed effects for *Eucalyptus* spp. included diameter, height, and age, while for *Pinus* spp., they were the Köppen climate classification, species, slenderness degree, and age. *R* estimates were mainly influenced by precipitation and age for both genera, with slenderness and diameter specifically affecting *Eucalyptus* spp., and height being a driving factor for *Pinus* spp. Our findings discourage the use of fixed or default values for BEF and *R* across locations with different climates and growing conditions to reduce uncertainties in carbon accounting and greenhouse gas inventories.

## Graphical abstract

This paper addresses the global greenhouse gas inventory challenge by reducing uncertainty and enabling more reliable carbon accounting to support strategic and policy decision‐making under climate variability.

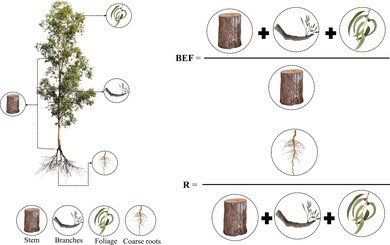

## 1 Introduction

Efforts to mitigate climate change impacts are increasingly a global priority, requiring urgent initiatives such as The Paris Agreement, adopted at the twenty‐first Conference of the Parties (COP 21) of the UNFCCC (United Nations Framework Convention on Climate Change), which mandates reductions in carbon emissions and enhancements in carbon sequestration (UNFCCC 2015; FAO 2016; Fisher 2024). A core commitment of the signatory countries is the annual reporting of national greenhouse gas (GHG) inventories, quantifying emissions and removals based on standardized methodologies under the UNFCCC and the Paris Agreement. These inventories follow the Intergovernmental Panel on Climate Change (IPCC) methodologies to ensure reliability, consistency, and comparability (IPCC 2006; Perugini et al. 2021). Additionally, countries are required to submit their nationally determined contributions (NDCs), outlining post‐2020 climate targets aimed at global decarbonization. These targets are expressed as total GHG emissions in carbon dioxide equivalent (GtCO_2_ ‐eq) (Denison et al. 2019; Siriwardana and Nong 2021; Sugsaisakon and Kittipongvises 2024). Nonetheless, current NDCs may be insufficient to keep global warming below 2°C or 1.5°C.

The forest sector plays a crucial role in achieving NDCs, serving as a key nature‐based solution (NbS) for carbon sequestration (Seddon et al. 2020; Li and Zhang 2024). In Brazil, forest plantations contribute significantly to GHG mitigation. Over 26 years, carbon sequestration by Brazilian forest plantations has offset emissions equivalent to those from the waste sector over the same period or from combined agriculture, forestry, and other land use sectors in 2016 (Sanquetta et al. 2018). Currently, production forests store 1.86 billion tons of CO_2_eq (IBÁ 2024). *Eucalyptus* and *Pinus* plantations have been key contributors, increasing their carbon storage by 165% (231 to 612 Mt) between 1990 and 2016 (Sanquetta et al. 2018).

Forests function both as carbon sinks and sources, mitigating climate change through CO_2_ sequestration and long‐term storage in biomass and soil (FAO 2016). Accurate quantification of above‐ and belowground biomass is essential for assessing carbon stocks. However, estimating forest biomass remains challenging due to the complexity of biological interactions in forest ecosystems (Sanquetta et al. 2011) and the historical focus of forest inventories on volume rather than biomass. Indirect biomass estimation methods include allometric equations and conversion factors, which facilitate biomass quantification using easily measurable field variables. Biomass expansion factors (BEF) and root‐to‐shoot ratios (*R*) are widely used due to their simplicity (Sanquetta et al. 2011; Brown 2002; Jagodziński et al. 2018). BEF represents the ratio of aboveground biomass to bole biomass, while *R* defines the ratio of root biomass to aboveground biomass.

A critical concern is the accuracy of generalized BEF and *R* values. Their applicability varies depending on species, growth stage, site index (Satoo 1982), climate (He et al. 2021), and specific conditions of the project (Sanquetta et al. 2011). The IPCC (2006, 2019) does not provide default BEF and *R* values for Brazil, nor for its predominant plantation genera, *Eucalyptus* and *Pinus*. The Ministry of Science, Technology, and Innovation (MCTI 2004) offers reference values for these plantations in Brazil, yet lacks transparency regarding sample size, regional representation, and age distribution, raising concerns about their applicability at a national scale.

To address these gaps, this study aims to: (i) develop BEF and *R* models for *Eucalyptus* and *Pinus* plantations, considering key influencing factors, and (ii) quantify differences between carbon estimates derived from these models and the default values from MCTI (2004). This study enhances understanding of biomass and carbon patterns, supporting the forest sector in improving carbon stock quantification and ensuring more accurate GHG inventories tailored to specific project conditions.

## 2 Materials and Methods

### 2.1 Description of the Sites

**FIGURE 1:**
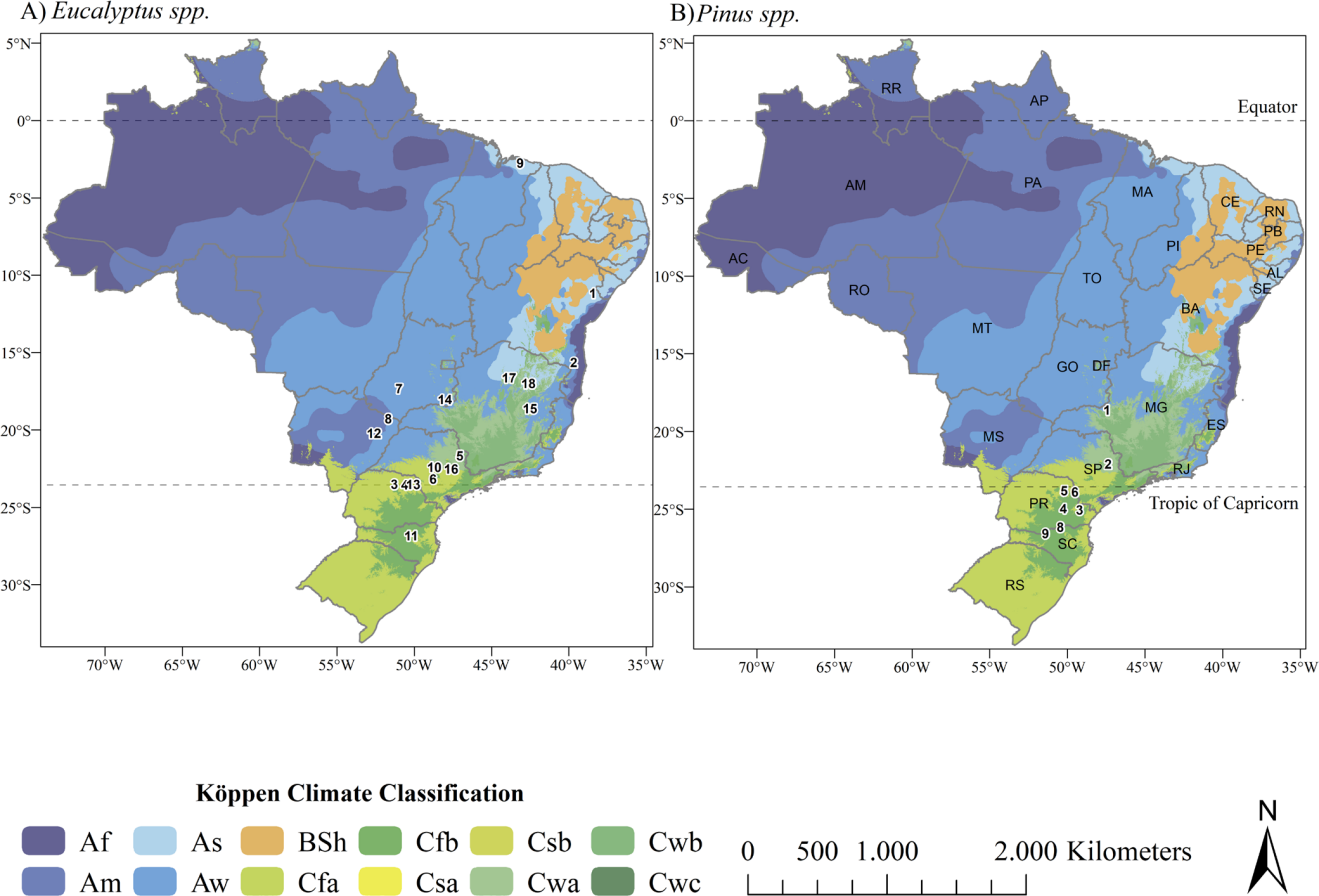
Locations of destructive biomass sampling sites across different climatic gradients in Brazil, for A) *Eucalyptus* spp. and B) *Pinus* spp. plantations. Map lines delineate study areas and do not necessarily depict accepted national boundaries.

This study utilized biomass data of *Eucalyptus* and *Pinus* plantations from different projects and research sites across a climatic gradient in Brazil, covering Maranhão to Santa Catarina states (Figure 1). The datasets comprise destructive sampling from 27 locations (18 for *Eucalyptus* spp., 9 for *Pinus* spp.) for aboveground biomass and 10 locations (8 for *Eucalyptus* spp., 2 for *Pinus* spp.) for belowground biomass (Table S1). These sites encompass around 60% of Brazil's Köppen climate classifications (Am, As, Aw, Cfa, Cfb, Cwa, and Cwb) and exhibit contrasting annual temperature (16.1°C–27.3°C) and precipitation (1045.1–1775.5 mm year^−1^) ranges (Alvares et al. 2013). The predominant soil types are Ferralsols (62% of the sites), followed by Acrisols (23%), Cambisols (12%), and Nitisols (4%), with texture classified as clay (69% of the sites) and sandy loam (31%).

### 2.2 Data Collection and Tree Database

The datasets primarily include aboveground biomass measurements (91.66%), covering the stem, dead branches, live branches, and leaves, compared to belowground biomass quantification (8.34%) for which coarse roots were considered. Trees selected for felling were systematically compartmentalized.

- *Stem*: woody material from the stump transition to the smallest branches, including bark.
- *Branches*: both live and dead woody material with bark.
- *Foliage*: all leaves.
- *Root systems*: coarse roots with a diameter greater than 10 Mm.

Fresh weights of aboveground components (stem, branches, foliage) were recorded in the field using a digital scale, and belowground biomass when possible. In the laboratory, representative samples were collected, labeled, and dried at 65°C to constant weight to determine dry mass. Stem samples were taken from 0%, 25%, 50%, 75%, and 100% of the commercial stem height (diameter 6–3 cm), including the tree top. Fresh weights were recorded using a semi‐analytical balance, and total dry weight (TDW) was estimated using Equation (1):

(1) $${\mathrm{TDW}}_{c}=\frac{{\mathrm{TFW}}_{c}*{\mathrm{DW}}_{s}}{{\mathrm{FW}}_{s}}$$

where TDW_c_ is the total dry weight of each tree component (stem, branches, foliage, roots), TFW_c_ is the total fresh weight of each tree component, DW_s_ is the dry weight of each sample, and FW_s_ is the fresh weight of each sample.

Total aboveground Equation (2) and belowground Equation (3) biomass was then calculated

(2) $${\mathrm{AGB}}_{\text{total}}=B_{\text{stem}}+B_{\text{branches}}+B_{\text{foliage}}$$

(3) $${\mathrm{BGB}}_{\text{total}}=B_{\text{roots}}$$

### 2.3 Biomass Expansion Factor and Root‐To‐Shoot Ratio Determination

The biomass expansion factor (BEF, dimensionless) is defined as the ratio of total aboveground dry biomass (AGB_total_ , kg tree^−1^) to stem dry biomass (B_stem_, kg tree^−1^) Equation (4). The root‐to‐shoot ratio (*R*, dimensionless) Equation (5) is established as the ratio of belowground dry biomass (BGB, kg tree^−1^) to aboveground dry biomass (AGB_total_ ). For this study, the total aboveground biomass comprises the entire tree, including tree tops, while the belowground biomass is limited to coarse roots.

(4) $$\mathrm{BEF}=\frac{{\mathrm{AGB}}_{\text{total}}}{B_{\text{stem}}}$$

(5) $$R=\frac{{\mathrm{BGB}}_{\text{total}}}{{\mathrm{AGB}}_{\text{total}}}$$

### 2.4 Modeling Approach

Data preprocessing included outlier detection per age class using the interquartile range (IQR) for *Eucalyptus* and *Pinus* response variables (BEF and *R*). Exploratory data analysis (EDA) was conducted to identify patterns in biomass distribution. Quantitative variables included stand age, climatological normals (average annual temperature and precipitation) (Alvares et al. 2013), dendrometric variables (total height, Ht, and diameter at breast height, dbh), and morphometric index (slenderness degree (S), calculated as the ratio between Ht and dbh). Qualitative variables encompassed genus (*Eucalyptus*, *Pinus*), Köppen climate classification, temperature classes, and age classes.

Model development progressed from simple linear regression and dummy variables to multiple linear regression, Lasso regression (Least Absolute Shrinkage and Selection Operator), and linear mixed‐effects models (LMMs). Initial models were fitted using the lm function (“stats” package, *R* v4.3.1), while LMMs were implemented using the lme function (“nlme” package), and Lasso regression was conducted using the glmnet function (“glmnet” package). Model performance was assessed via *k*‐fold cross‐validation (*k* = 10), portioning the dataset into training and validation subsets to mitigate overfitting and underfitting (Jiang and Chen 2016; Tchakoucht et al. 2024). Robustness was assessed through heteroscedasticity (Breusch–Pagan test), normality (Shapiro–Francia test), and multicollinearity (variance inflation factor–VIF < 10).

### 2.5 Goodness‐of‐Fit Metrics for Evaluating Model Performance

To ensure the reliability and accuracy of the developed models, we assessed their performance using a suite of statistical metrics. These metrics allowed for a comprehensive evaluation of how well the models fit the observed data and their generalization capability when applied to new datasets. The assessment included both traditional goodness‐of‐fit measures and cross‐validation techniques.

Model selection was guided by Taylor's diagram, which provides a graphical representation of model performance by statistics, including correlation coefficient, standard deviation, and root mean square error (Taylor 2001). This visualization facilitated the comparison of multiple models and helped identify the most robust and parsimonious approach. Additionally, the following statistical metrics were employed: Akaike information criterion (AIC, Equation (6)), fit index (FI, Equation (7)), root mean square error in absolute terms (RMSE, Equation (8)) and in percentage (RMSE%, Equation (9)), and mean absolute error (MAE, Equation (10)). To further validate model reliability, we applied residual analysis, including residual plots and histograms of percentage error (%) Equation (11), in which *k* is the number of parameters in the model, *n* is the total number of observations, $Y_{i}$ is the measured value of the response variable in the *ith* observation. ${\hat{Y}}_{i}$ is the predicted value of the response variable in the *ith* observation, and ${\bar{Y}}_{i}$ is the average value of the response variable.

(6) AIC=−2loglik+2k

(7) $$\mathrm{FI}=1-\frac{\sum_{i=1}^{n}{\left(Y_{i}-{\hat{Y}}_{i}\right)}^{2}}{\sum_{i=1}^{n}{\left(Y_{i}-{\bar{Y}}_{i}\right)}^{2}}$$

(8) $$\text{RMSE}=\sqrt{\frac{\sum_{i=1}^{n}{\left(Y_{i}-{\hat{Y}}_{i}\right)}^{2}}{n}}$$

(9) $$\text{RMSE}\%=\frac{100}{\bar{Y}}\sqrt{\frac{\sum_{i=1}^{n}{\left(Y_{i}-{\hat{Y}}_{i}\right)}^{2}}{n}}$$

(10) $$\mathrm{MAE}=\frac{\sum_{i=1}^{n}\left|Y_{i}-{\hat{Y}}_{i}\right|}{n}$$

(11) $$E\text{rror}\left(\%\right)=\frac{\left(Y_{i}-{\hat{Y}}_{i}\right)}{Y_{i}}100$$

### 2.6 Benchmarking Analysis of the Quality of the BEF and *R* Values for *Eucalyptus* and *Pinus* Plantations in Brazil

This final step consisted of a benchmarking analysis of the BEF and *R* values obtained using different approaches to assess their quality, accuracy, and representativeness in *Eucalyptus* and *Pinus* plantations in Brazil at two scales: experimental and commercial. The objective was to evaluate how well the obtained values align with the standard references used in Brazil's national greenhouse gas inventory and forestry sector reports.

At the experimental scale, analyses were conducted at the same sites used in this study. At the commercial scale, analyses were performed at the national level, encompassing the entire *Eucalyptus* and *Pinus* plantation area in Brazil as of 2022, based on a spatial dataset provided by Suzano SA. This dataset comprises 7.54 million hectares of *Eucalyptus* plantations and 1.78 million hectares of *Pinus* plantations and includes estimated planting dates. Biomass estimation for commercial stands nationwide was conducted using height and diameter equations as a function of age, which were calibrated based on tree measurements from the study sites and applied to each stand in the national geodatabase (Table S3).

Thus, we assumed BEF (*Eucalyptus* spp.: 1.20, and *Pinus* spp.: 1.25), and *R* (0.35 for both genera) default values for Brazil based on MCTI (2004). Therefore, the following comparisons (Equation (12 up to 15)) were established in percentage terms (Dif%), in which y_d_ is Brazil's default based on MCTI value for BEF or *R*, y_r_ is observed BEF or *R* values for each temperature or age class, y_p_ is predicted BEF or *R* values from models developed in this study for each temperature or age class, and ȳ is the general average of BEF and *R* for each genus obtained from the data observed in the sampling of this study.

(12) $${\mathrm{Dif}}_{\text{MCTI}\times \text{Observed}}\left(\%\right)=\frac{y_{d}-y_{r}}{y_{r}}100$$

(13) $${\mathrm{Dif}}_{\text{MCTI}\times \text{Predicted}}\left(\%\right)=\frac{y_{d}-y_{p}}{y_{p}}100$$

(14) $${\mathrm{Dif}}_{\text{Study average}\times \text{Observed}}\left(\%\right)=\frac{\bar{y}-y_{r}}{y_{r}}100$$

(15) $${\mathrm{Dif}}_{\text{Predicted}\times \text{Observed}}\left(\%\right)=\frac{y_{p}-y_{r}}{y_{r}}100$$

## 3 Results

### 3.1 Descriptive Analysis

Given the easiest and cheapest method of collecting aboveground biomass, there was a greater representation of BEF (91.7%) in the database compared to *R* (8.3%), which requires the collection of coarse roots. In terms of genera, there was also a greater sampling of *Eucalyptus* spp. (89.9%) compared to *Pinus* spp. (10.1%) due to the greater availability of data on *Eucalyptus* species, which are the most widely planted in Brazil. The *Pinus* spp. sampling data covered the regions that account for more than 80% of the *Pinus* plantation area in Brazil. Generally, age was the variable with the highest variability across the entire dataset, followed by dbh, concerning BEF, while Ht showed the greatest variability with regard to *R*. *Eucalyptus* spp. showed less variation in terms of BEF and *R*, despite being situated within a stronger climatic gradient than *Pinus* spp. *Eucalyptus* spp. compared to *Pinus* spp. showed an increase in the coefficient of variation (CV%) for meteorological variables by an average of 34% and 82% in the BEF and *R* sampling regions, respectively. Additionally, *Eucalyptus* spp. exhibited greater genetic variability than *Pinus* spp. The coefficients of variation for BEF and *R* were 8.9% and 17.8% lower, respectively, for *Eucalyptus* spp. compared to *Pinus* spp. (Table 1).

**TABLE 1:** Descriptive analysis of the database for BEF and *R* for *Eucalyptus* and *Pinus*.

Genera	Biomass expansion factor (BEF)								
	*n*	Variables/statistics	Age (years)	dbh (cm)	Ht (m)	S	T (°C)	P (mm)	BEF
*Eucalyptus* spp.	1843	Min.	2.0	3.21	6.0	0.81	16.07	1045.12	1.01
		Mean	4.69	13.89	21.17	1.55	20.45	1354.15	1.16
		Max.	8.4	28.2	35.7	2.35	27.32	1775.53	1.63
		Std. Dev.	1.73	4.21	6.02	0.23	2.21	150.16	0.13
		CV	36.9	30.3	28.4	14.84	10.8	11.1	11.2
*Pinus* spp.	190	Min.	7.3	9.99	8.8	0.48	15.47	1280.71	1.03
		Mean	11.62	21.88	17.89	0.84	18.32	1479.7	1.22
		Max.	17.3	34.7	27	1.66	20.46	1747.33	1.7
		Std. Dev.	2.96	5.25	4.14	0.18	1.38	131.48	0.15
		CV	25.5	24.0	23.1	21.4	7.5	8.9	12.3

Genera	Root‐to‐shoot ratio (*R*)								
	*n*	Variables/statistics	Age (years)	dbh (cm)	Ht (m)	S	T (°C)	P (mm)	*R*
*Eucalyptus* spp.	150	Min.	2.3	7.0	10.6	0.89	18.65	1045.12	0.10
		Mean	4.41	14.57	20.77	1.44	20.18	1311.58	0.22
		Max.	8.5	26.6	34.1	1.96	24.68	1456.19	0.49
		Std. Dev.	1.82	3.7	5.71	0.23	1.49	125.28	0.07
		CV	41.3	25.4	27.5	15.9	7.4	9.6	31.8
*Pinus* spp.	35	Min.	7.3	12.99	8.8	0.49	18.65	1280.71	0.16
		Mean	10.67	20.98	16.07	0.77	19.11	1401.35	0.31
		Max.	17.3	29.5	24.95	1.26	20.46	1443.11	0.62
		Std. Dev.	3.78	4.12	4.44	0.17	0.81	72.01	0.12
		CV	35.4	19.6	27.6	22.1	4.2	5.1	38.7

*Note: n* is the sample size, BEF is the biomass expansion factor, *R* is the root‐to‐shoot ratio, dbh is the diameter at 1.30 m aboveground, Ht is total height, S is slenderness degree, T is the average temperature, P is precipitation, Std. Dev. is the standard deviation, CV is the coefficient of variation (%), Min. is the minimum value of a variable, and Max. is the maximum value of a variable.

**FIGURE 2:**
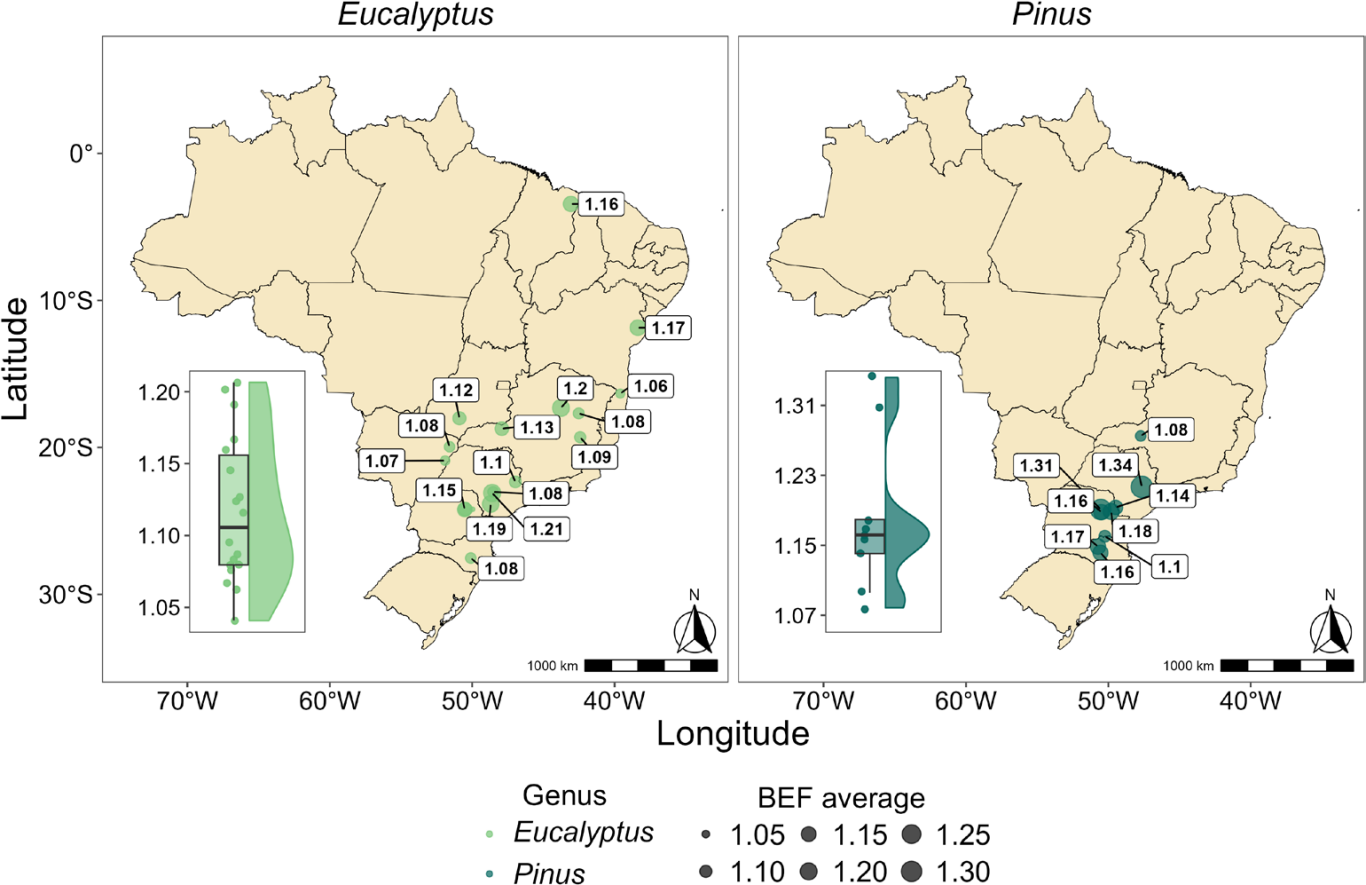
Map of the average biomass expansion factor (BEF) values across Brazil. Map lines delineate study areas and do not necessarily depict accepted national boundaries.

**FIGURE 3:**
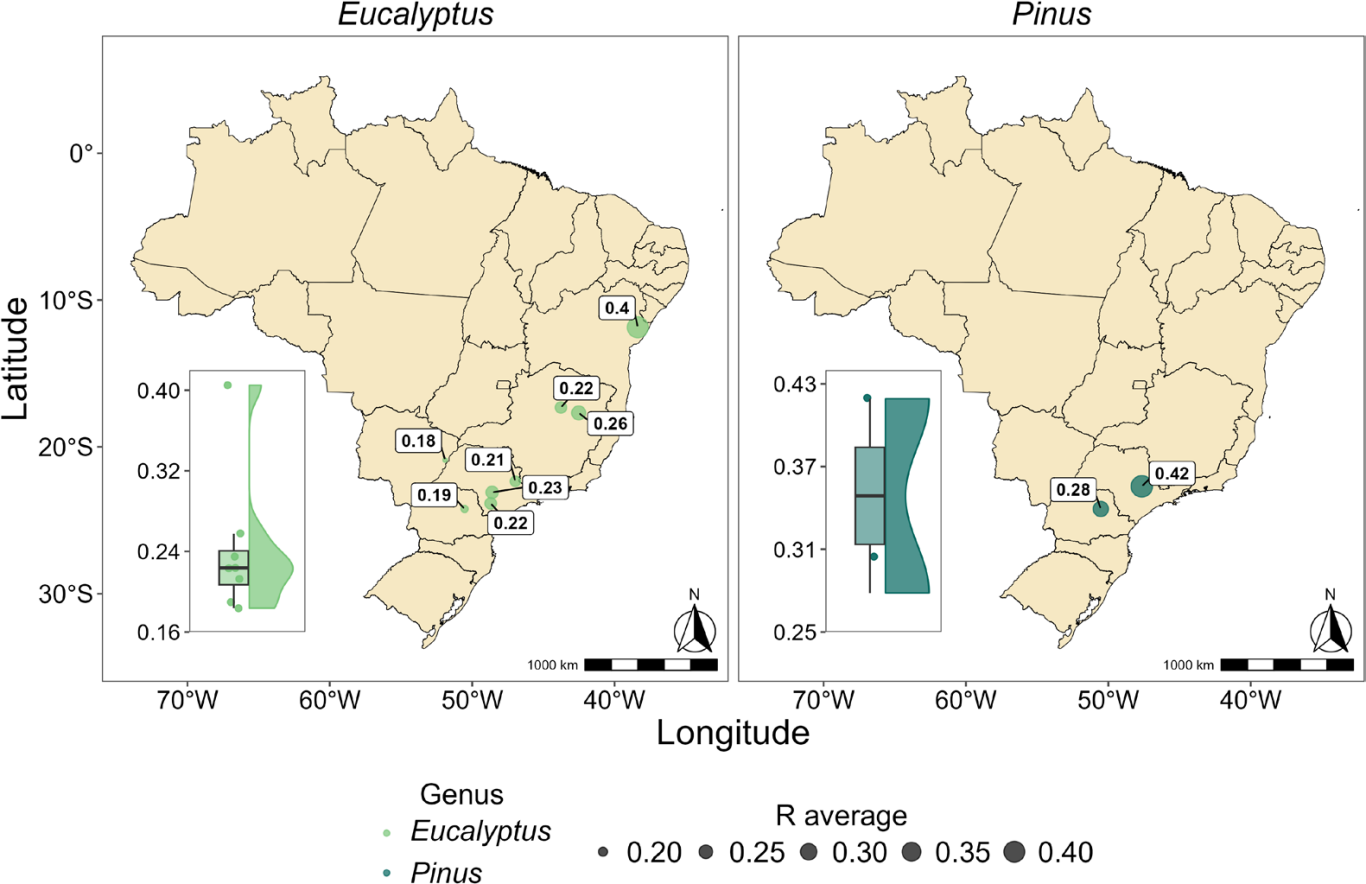
Map of the average root‐to‐shoot ratio (*R*) across Brazil. Map lines delineate study areas and do not necessarily depict accepted national boundaries.

Figure 2 clearly illustrates that the *Eucalyptus* spp. samples were within a broader climatic gradient than those of the *Pinus* spp. However, the BEF values for *Eucalyptus* spp. had a lower regional range of variation (0.17) than those of the *Pinus* spp. (0.26), with a percentage difference of 52.9%. The highest BEF values for both genera occurred in São Paulo state, characterized by the Köppen Cfa climate (Figure 2). In terms of *R*, a greater amplitude of this variable was observed for *Eucalyptus* spp. (0.22) compared to *Pinus* spp. (0.14) (Figure 3). The highest *R* values for both genera were similar, occurring in regions with higher temperatures and lower precipitation within the sample; these values were observed in Bahia for *Eucalyptus* spp. and São Paulo for *Pinus* spp. It is important to highlight that these regions include samples of different ages and genetic materials, which may also influence the variation in biomass expansion factors (BEF) and root‐to‐shoot ratios (*R*).

**FIGURE 4:**
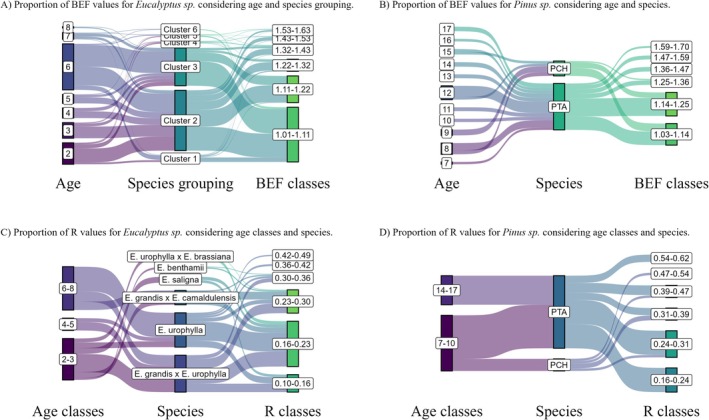
Description of BEF and *R* based on a Sankey diagram for *Eucalyptus* and *Pinus* at different ages. Panels A–B present the BEF analysis for *Eucalyptus* and *Pinus* plantations, respectively, while panels C–D display the R analysis for *Eucalyptus* and *Pinus* plantations. Species grouping according to the biomass expansion factor (BEF) for *Eucalyptus* (see details in Figure S1): Cluster 1 ( *E. saligna* , and *E. benthamii* ), cluster 2 ( *E. tereticornis* , *E. grandis* , *E. dunnii* , and *E. grandis* × *E. urophylla*), cluster 3 (*E. longirostrata*, *E. grandis* × *E. camaldulensis* , and *E. urophylla*), cluster 4 ( *E. pilularis* , and *E. pelita*), cluster 5 (*E. urophylla* × *E. brassiana*), and cluster 6 (undefined genetic material).

In general, the number of BEF samples for *Eucalyptus* spp. is concentrated at 6 years of age, followed by ages 2 and 3 (Figure 4A). For a better graphical visualization, the *Eucalyptus* species/hybrids were grouped based on the similarity of their BEF values (detailed in Figure S1). Clusters 2 and 3 (Figure 4A) cover a wide age range with predominance at age 6. These clusters contribute to a greater proportion of the smaller BEF classes. However, cluster 2 encompasses a greater range of BEF classes, which may reflect its sampling, since it also includes a greater age range (Figure 4A). *Pinus taeda* (PTA) is the predominant *Pinus* species in the database, covering a wider range in terms of age with a predominance of ages over 12 years. In contrast, *Pinus caribaea* var. *hondurensis* (PCH) has greater representation at younger ages (8 and 9 years). Furthermore, it is noted that *Pinus taeda* has higher BEF values compared to *Pinus caribaea* var. *hondurensis* (Figure 4B).

*R* data for *Eucalyptus* species/hybrids reveal that the older (6–8) and younger (2–3) age classes are the most representative of the data, with a higher proportion of *E. urophylla* and *E. grandis* × *E. urophylla*. These species also stand out for their largest proportion in this sample. Notably, *E. urophylla*, *E. grandis* × *E. urophylla*, *E. grandis* × *E. camaldulensis* , and *E. benthamii* reached high *R* values (above 0.30) even though they were presented in smaller proportions. However, *R* values lower than or equal to 0.30 occur in greater proportions, typically from *E. urophylla*, *E. grandis* × *E. urophylla*, and *E. grandis* × *E. camaldulensis* (Figure 4C). Regarding the *Pinus* species, PCH varies by 42.9% in terms of age and by 125% in terms of *R* values. PTA comprises approximately 8 distinct ages and varies by 287.5% in terms of *R* values, although its greatest concentration occurs in the lowest *R* classes (≤ 0.31) (Figure 4D).

**FIGURE 5:**
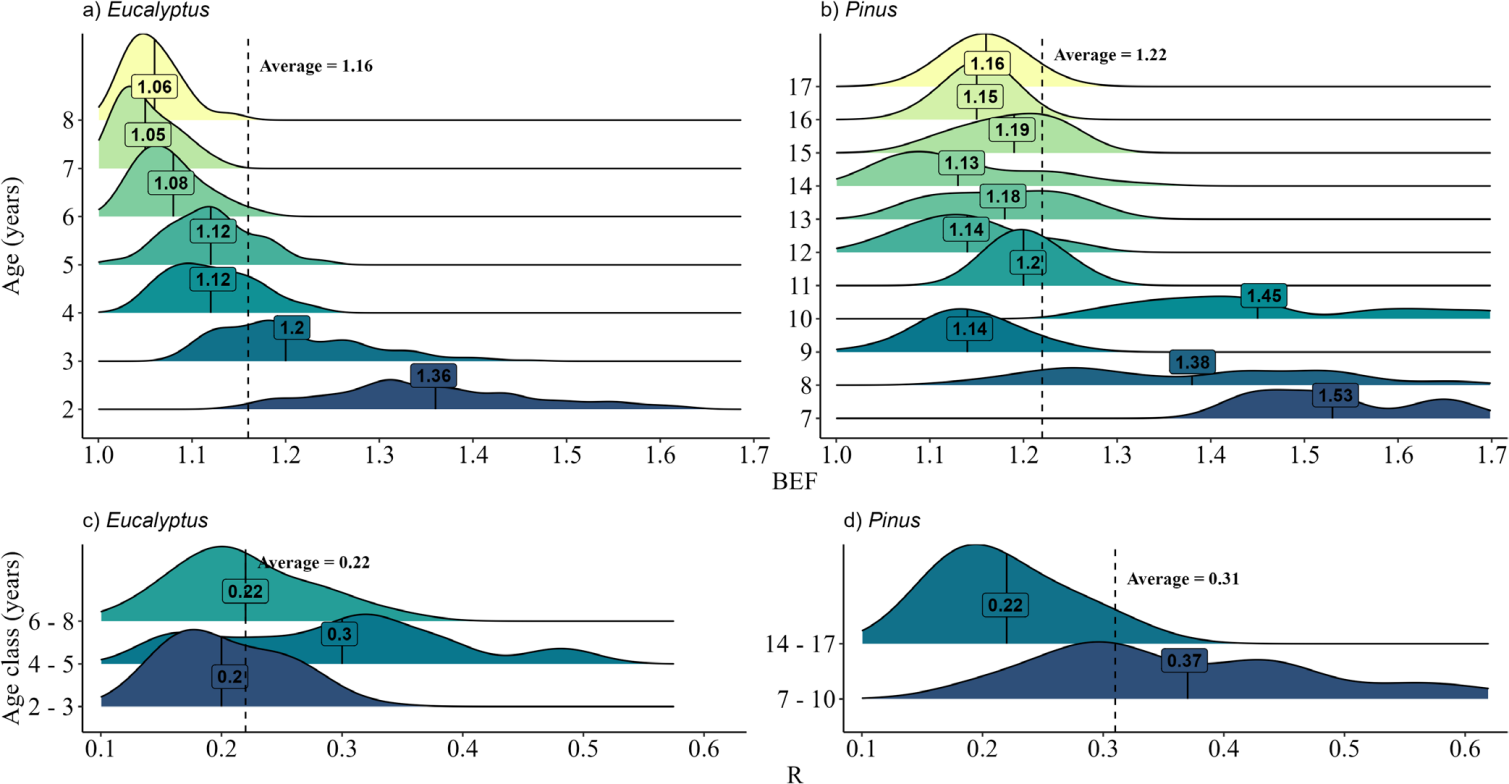
Distribution analysis of the biomass expansion factor (BEF) and root‐to‐shoot ratio (*R*) values according to age and genera. Panels A–B present the BEF analysis for *Eucalyptus* and *Pinus* plantations, respectively, while panels C–D display the R analysis for *Eucalyptus* and *Pinus* plantations, respectively.

It is important to highlight that the distribution of the BEF and *R* data reflects the diversity of the projects we are analyzing since we did not perform targeted sampling to represent the average BEF and *R* values for each species at different ages and locations. We integrated databases from several projects with different purposes. This is a crucial aspect in understanding that the heterogeneity of the data sources can largely influence BEF and *R* distributions. In general, these distributions were skewed to the right, indicating that the BEF and *R* variables had relatively low values; however, higher values influenced their averages (Figure 5). Specifically for *Eucalyptus* spp., a clear pattern of a decrease in BEF of approximately 22.8% occurred with increasing age, indicating a greater biomass accumulation in the stem with tree age. On the other hand, for *Pinus* spp., the BEF values were higher at ages 7, 8, and 10 years, whereas at the remaining ages, the values were below the mean. The varied pattern of BEF distribution for *Pinus* spp. reflects the distinct behavior of the two species included in this study, *Pinus taeda* and *Pinus caribaea* var. *hondurensis*. It is worth noting that, at 9 years of age, only *Pinus caribaea* var. *hondurensis* samples were available, which may have been a determining factor in the sudden decrease in BEF. In terms of *R*, there was a 40.5% reduction in this factor for *Pinus* spp. with increasing age classes, as opposed to *Eucalyptus* spp., where there was a 50% increase between the 2–3 and 4–5 year age classes, followed by a 26.7% reduction for the highest age class (6–8 years).

### 3.2 Modeling the Biomass Expansion Factor (BEF) and Root‐To‐Shoot Ratio (*R*)

In BEF modeling, the simple and multiple linear regression models did not satisfy the statistical assumptions of normality and homoscedasticity of the residuals, requiring the implementation of more complex models, which included linear mixed models and lasso regression. The Taylor diagram (Figure S2) reveals that both models were suitable for modeling the BEF for both genera, with the LMMs demonstrating a slight superiority in predictive performance. This fact justifies the selection of mixed models in this study since they more accurately captured the variations in the data for each genus than lasso regression. Additionally, the choice of LMMs to model BEF was also motivated by the hierarchical structure of the dataset, which includes a large number of repeated measurements and allows us to account for both fixed and random effects associated with grouping structures (age and temperature classes), improving the generalizability and robustness of the model.

Corroborating the selection of mixed models, the statistics show good predictive performance for both genera with FI values around 0.8 and low error metrics (RMSE and MAE) (Table 2). Based on the intraclass correlation coefficient (ICC) values, the success of these models in terms of accuracy can be attributed mainly to the random effect on the intercept (Equation (16) and Table S1) determined by the mean temperature classes that explained 99.2% of the BEF variation for *Eucalyptus* spp. and by the age classes that captured 88.3% of the variation in BEF behavior for *Pinus* spp. Equation (17). It is noted that the fixed effect parameters also varied according to the genera, with predominantly dendrometric variables being significant for *Eucalyptus* spp., whereas for *Pinus* spp. the interaction of the variables was decisive in capturing BEF patterns, for example, species × Köppen climate classification, in addition to the slenderness degree × age.

(16) $${\mathrm{BEF}}_{\mathrm{ij}}=\left(\beta_{0}+b_{0j}\right){\mathrm{TC}}_{\mathrm{ij}}+\beta_{1}\frac{1}{{\mathrm{dbh}}_{\mathrm{ij}}}+\beta_{2}\ln\left({\mathrm{Ht}}_{\mathrm{ij}}\right)+\beta_{3}\frac{1}{{\mathrm{Age}}_{\mathrm{ij}}}++b_{1j}{\mathrm{Ht}}_{\mathrm{ij}}+\varepsilon_{\mathrm{ij}}$$

(17) $${\mathrm{BEF}}_{\mathrm{ij}}=\left(\beta_{0}+b_{0j}\right){\mathrm{AC}}_{\mathrm{ij}}+\beta_{1}{\text{Köppen}}_{\mathrm{ij}}:{\mathrm{SP}}_{\mathrm{ij}}+\beta_{2}S_{\mathrm{ij}}:\frac{1}{{\mathrm{Age}}_{\mathrm{ij}}}+\varepsilon_{\mathrm{ij}}$$

where BEF_ij_ is the biomass expansion factor (dimensionless), TC_ij_ is the temperature class, dbh_ij_ is the diameter at 1.30 m aboveground (in centimeters), Age_ij_ is the stand age (in years), Ht_ij_ is the total height (in meters), AC_ij_ is the age class, Köppen_ij_ is the Köppen climate classification, SP_ij_ is a dummy variable for species (1– *Pinus caribaea* var. *hondurensis*, and 0– *Pinus taeda* ), *S*_ij_ is the slenderness degree, *β*_i_ represents fixed coefficients, b_0j_ and b_1j_ are random coefficients based on temperature classes and total height for *Eucalyptus* spp. and age classes for *Pinus* spp., and subscripts *i*, *j* refer to the individual tree *i* of temperature class *j* or age class *j* according to the genera; *ε*_ij_ *N* (0, σ^2^I) and *b*_j_ *N* (0, *σ* ^2^D).

**TABLE 2:** Parameter estimates and goodness‐of‐fit statistics from ten‐fold evaluation for linear mixed‐effects models used in the biomass expansion factor (BEF) predictions.

Mixed‐effects model								
Fixed effects	Estimate	Std. Error	*t*‐value	Random effects	Variance		Std. Dev.	Corr
*Eucalyptus* spp.								
Intercept	4.00485	0.14187	28.23427	Intercept	0.5344		0.73086	−1
1/dbh	−2.30280	0.11611	−19.8340	Ht	0.00343		0.05853	
Ln (Ht)	−0.90868	0.04281	−21.22970	Residual	0.00096		0.03105	
1/age	0.59804	0.02564	23.32938					
*Pinus* spp.								
Intercept	1.40926	0.06234	22.61034	Intercept	0.03096		0.17592	—
Cfa:SP	−0.22564	0.02439	−9.25902	Residual	0.00409		0.06394	
Cfb:SP	−0.36197	0.03189	−11.38867					
Cwb:SP	−0.03912	0.02175	−1.79902					
S:(1/age)	−2.16038	0.39088	−5.52546					
Ten‐fold validation statistics								
Genera				FI	RMSE	RMSE%	MAE	AIC
*Eucalyptus* spp.				0.81	0.059	5.035	0.041	−4591.26
*Pinus* spp.				0.80	0.064	5.277	0.051	−379.44

*Note:* dbh is the diameter at 1.30 m aboveground, Ht is the total height, S is the slenderness degree, SP is a dummy variable for species (1– *Pinus caribaea* var. *hondurensis* and 0– *Pinus taeda* ), Ln is the natural logarithm, Std. Error is the standard error, Std. Dev. is the standard deviation, Corr is the correlation between the intercept and slope, and the symbol: indicates an interaction.

On the other hand, for the response variable *R*, LMMs were not applied due to the limited number of observations and the absence of clear hierarchical or nested data structures. In this case, introducing random effects would lead to overparameterization without a meaningful gain in model performance. Moreover, exploratory analysis indicated that a linear structure sufficiently captured the observed variation in *R* concerning the predictor variables. Therefore, the *R* modeling for both genera used simpler approaches than BEF modeling, as the data also met the assumptions of classical linear regression, i.e., normality and homoscedasticity of the residuals. Dummy variables were used for *Eucalyptus* spp., with age classes coded as 1 (less than or equal to 3 years) or 0 (greater than 3 years), and the response variable was log‐transformed for both genera. All parameters of the adjusted models were statistically significant at the 1% level for Student's *t*‐test. Precipitation and age influenced the estimates of *R* for both genera, along with the interactions of age (under different types of transformation) with the slenderness degree and diameter for *Eucalyptus* spp. Equation (18) and with total height for *Pinus* spp. Equation (19). In modeling terms, *R* had a lower predictive performance than BEF, which can be explained by the limited number of samples used to develop the models. Even so, the models explained more than 50% of the variation in the data and resulted in acceptable errors, highlighting the superior predictive performance for *Pinus* spp. (Table 3).

(18) $$\ln\left(R_{i}\right)=\beta_{0}+\beta_{1}\left({\mathrm{Age}}_{i}^{2}.{\mathrm{dbh}}_{i}\right)+\beta_{2}\left(\frac{1}{P_{i}}\right)+\beta_{3}\left({\mathrm{AC}}_{i}:S_{i}\right)+\varepsilon_{i}$$

(19) $$\ln\left(R_{i}\right)=\beta_{0}+\beta_{1}P_{i}+\beta_{2}\left(\frac{1}{{\mathrm{Age}}_{i}}\right)+\beta_{3}\left({\mathrm{Age}}_{i}.{\mathrm{Ht}}_{i}\right)+\varepsilon_{i}$$

where *R*_i_ is the root‐to‐shoot ratio (dimensionless), dbh_i_ is the diameter at 1.30 m aboveground (in centimeters), Age_i_ is the stand age (in years), *P*_i_ is the precipitation (in millimeters year^−1^), Ht_i_ is the total height (in meters), AC_i_ is the dummy variable for age classes (1–less than or equal to 3 years or 0–greater than 3 years), *S*_i_ is the slenderness degree, *β*_i_ represents fixed coefficients, subscript *i* refers to individual tree *i*, and ε_i_ is the random error.

**TABLE 3:** Parameter estimates and goodness‐of‐fit statistics for the evaluation of linear regression models used in root‐to‐shoot ratio (*R*) predictions.

Coefficients	Estimate	Std. Error	*t* value		Pr (>|t|)
*Eucalyptus* spp.—dummy variable model					
Intercept	−2.432058	0.1746	−13.932		< 2e‐16***
Age^2^*dbh	−0.0005827688	7.881e‐05	−7.395		1.02e‐11***
1/P	1683.687	210.6	7.994		3.63e‐13***
AC: S	−0.3531248	0.03883	−9.093		6.43e‐16***
*Pinus* spp.—multiple linear regression					
Intercept	5.041	1.216	4.146		0.000243***
P	−0.002886	0.0006252	−4.616		6.44e‐05***
1/Age	−12.82	3.602	−3.560		0.001221**
Age*Ht	−0.00481	0.0009091	−5.290		9.34e‐06***
Statistics on the quality of model estimates					
Genera	*R* ^2^ adjusted	RMSE	RMSE%	MAE	AIC
*Eucalyptus* spp.	0.51	0.044	19.631	0.035	−60.48
*Pinus* spp.	0.66	0.069	22.078	0.052	−4.91

*Note:* dbh is the diameter at 1.30 m aboveground, Ht is the total height, AC is a dummy variable for age classes (1–less than or equal to 3 years or 0–greater than 3 years), P is the precipitation, S is the slenderness degree, Std. Error is the standard error. The symbols: and * mean interaction and multiplication, respectively. The asterisks *** and ** indicate the significance of parameters, respectively, at 1% and 5% levels.

**FIGURE 6:**
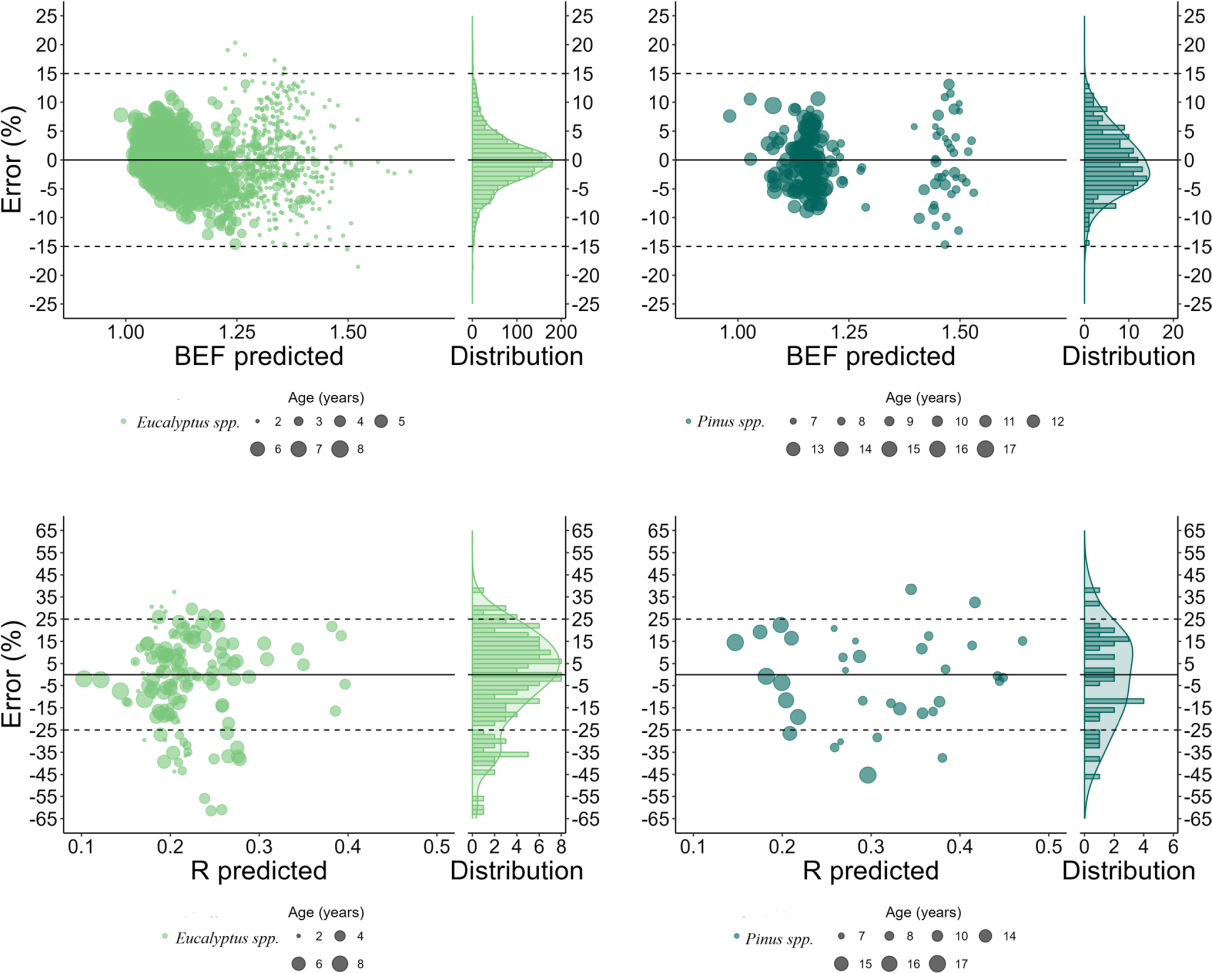
Scatter plots with marginal residual histograms of the models selected in the BEF and *R* predictions.

Complementary to the statistics, the graphical analysis of the residuals demonstrated that the errors in the BEF estimates were concentrated between 15% and 15% for both genera, following a normal distribution in terms of residual frequency (greater concentration on the 0 axis) for *Eucalyptus* spp. and with slight asymmetry for *Pinus* spp. Notably, the largest errors were attributable to the estimates at the youngest ages. Regarding the *R* estimates, there was a greater error amplitude, which was concentrated in the range between 25% and 25% for both genera with slight asymmetry in the distribution of errors with a tendency towards overestimation, regardless of age (Figure 6).

### 3.3 Quality Analysis of the Biomass Expansion Factor (BEF) and Root‐To‐Shoot Ratio (*R*) for *Eucalyptus* and *Pinus* Plantations in Brazil

**FIGURE 7:**
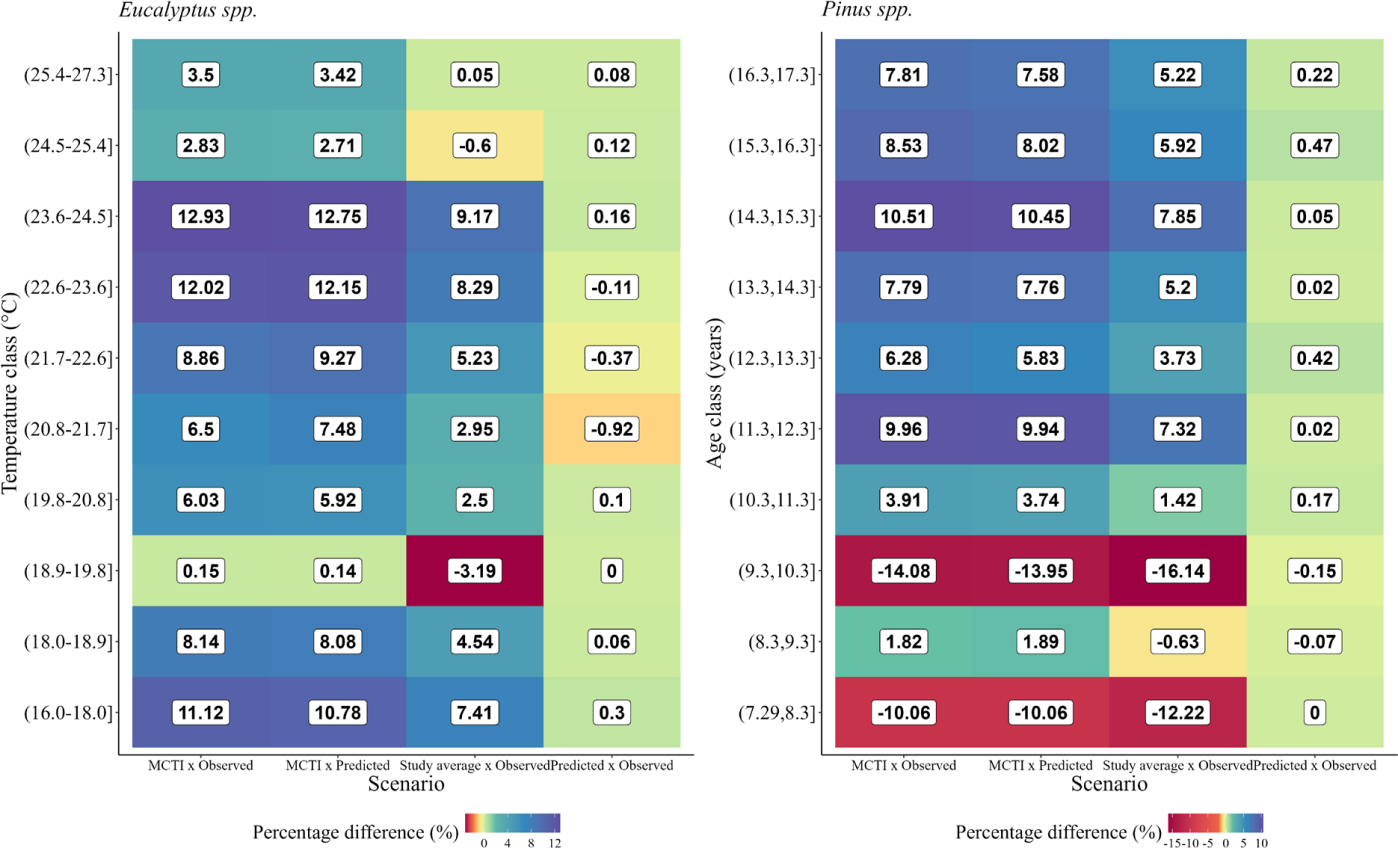
Biomass expansion factor (BEF) scenarios of the percentage difference for *Eucalyptus* spp. and *Pinus* spp.: (i) default BEF from the Ministry of Science, Technology, and Innovation – MCTI versus observed BEF sampled in this study, (ii) MCTI default BEF versus BEF predicted using linear mixed models, (iii) average BEF of this study versus observed BEF, and (iv) BEF predicted using linear mixed models versus observed BEF.

In BEF terms, the random effects of the mixed modeling were considered the main drivers of biomass allocation. Therefore, the temperature and age classes for *Eucalyptus* spp. Equation (12) and *Pinus* spp. Equation (13), respectively, were used to generate average values that were compared to real values and MCTI (2004) default values in the benchmarking analysis (Figure 7). Using the MCTI default value for *Eucalyptus* spp. evidences a tendency to overestimate the real data by up to 12.93%. Overall, the average observed in this study reveals the same trend, although with lower errors than the MCTI, as 9.17% is the largest deviation recorded. The largest deviations of the abovementioned scenarios occur for the 22.6°C–23.6°C and 23.6°C–24.5°C temperature classes. The study average was worse than the MCTI default only for the 18.9°C–19.8°C temperature class, for which the model was the most accurate, with an error close to zero. The sampling within this temperature class was predominantly from the Cfa Köppen climate, representing 54.64% of the data (Table S1). Notably, the BEF model estimates followed a similar pattern to that observed in the real data regarding their differences from the MCTI default. The reliability of these estimates concerning field reality was shown by their minimal discrepancy, which reached a maximum of 1%.

For *Pinus* spp., the MCTI default value tended to overestimate the real data; however, the largest deviation was an underestimation of approximately 14% for the 9.3–10.3 year age class. The average BEF from this study for *Pinus* spp. also indicated a similar tendency to the MCTI default value to overestimate the real data, although with lower deviations. Only in the 7.29–8.3 and 9.3–10.3 year age classes was this study's average worse than the MCTI default value, denoting a tendency toward underestimation. These findings indicate that the BEF estimates based on dendrometric attributes, species, and climate variables are more consistent with the field reality than fixed values, as evidenced by errors under 0.5%.

**FIGURE 8:**
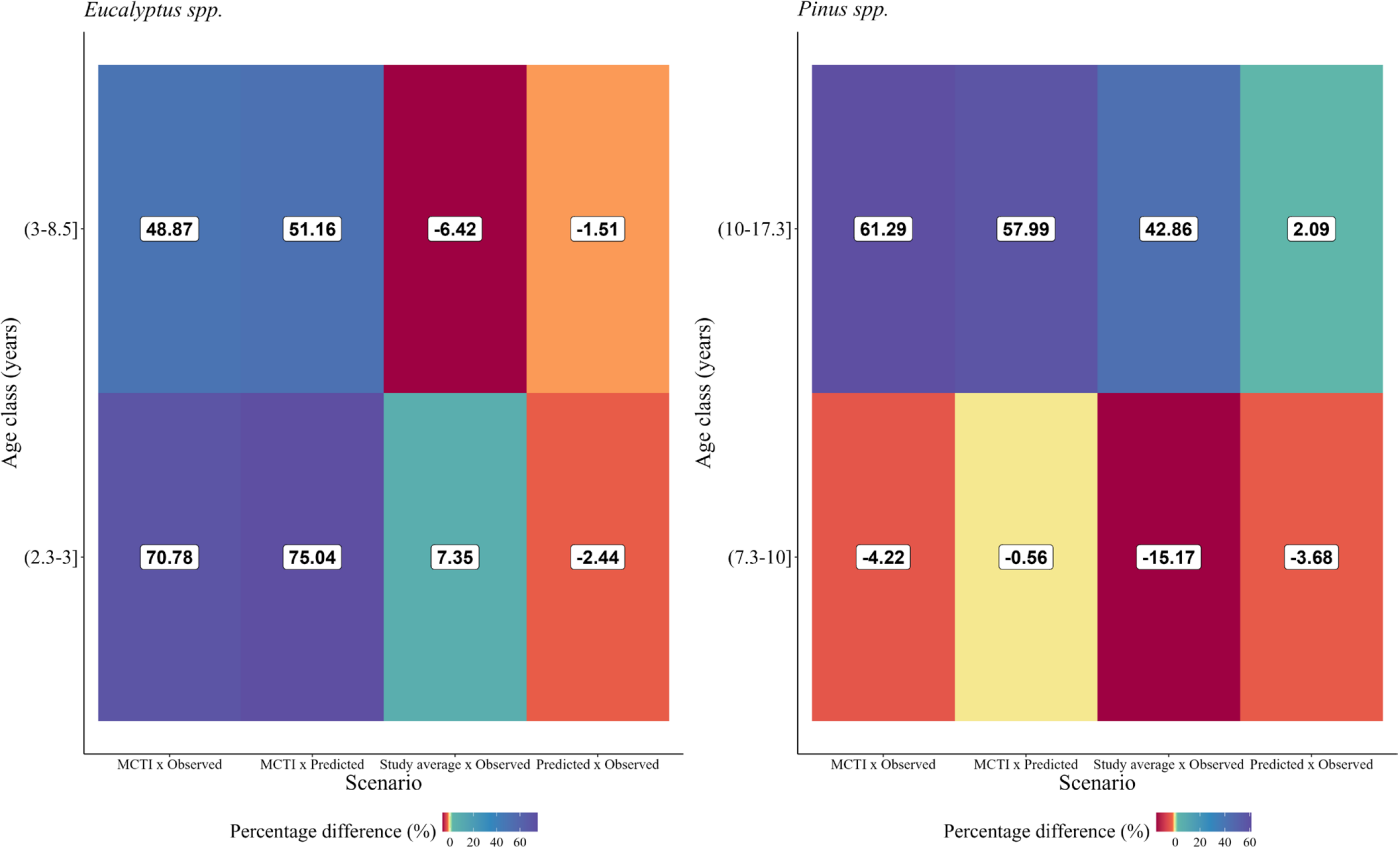
Root‐to‐shoot ratio (*R*) scenarios of the percentage difference for *Eucalyptus* spp. and *Pinus* spp.: (i) default *R* from the Ministry of Science, Technology, and Innovation—MCTI versus observed *R* obtained in this study, (ii) MCTI default *R* versus *R* predicted using linear models, (iii) average *R* of this study versus observed *R*, and (iv) *R* predicted using linear models versus observed *R*.

Benchmarking analysis of *R* values was conducted based on average values by age classes for both genera (Figure 8). MCTI default values had a strong trend of overestimation, with an average of 60.3% for both genera, except for the youngest individuals (7.3–10 years) of *Pinus* spp. for which there was a slight underestimation. The use of a fixed value, even when based on the average *R* obtained in this study for both genera, also deviated from field observations. However, the study average exhibited slightly smaller deviations than the MCTI default value, except for *Pinus* spp. in the age range of 7.3–10 years. In contrast, using *R* values that vary at the tree level, derived from the models proposed in this study, more accurately captured the behavior of the observed data, with an error of less than 4% for both genera.

### 3.4 Scaling the Biomass Expansion Factor (BEF) and Root‐To‐Shoot Ratio (*R*) for *Eucalyptus* and *Pinus* Plantations at the Commercial Scale in Brazil

To upscale the BEF and *R* factors to a commercial scale, the adjusted models were employed. These models used to estimate the dendrometric variables DBH and total height as a function of age served as inputs for the BEF and *R* models developed in this study. All models met the assumption of variance homogeneity, with all parameters statistically significant at the 5% level (Table S3).

**FIGURE 9:**
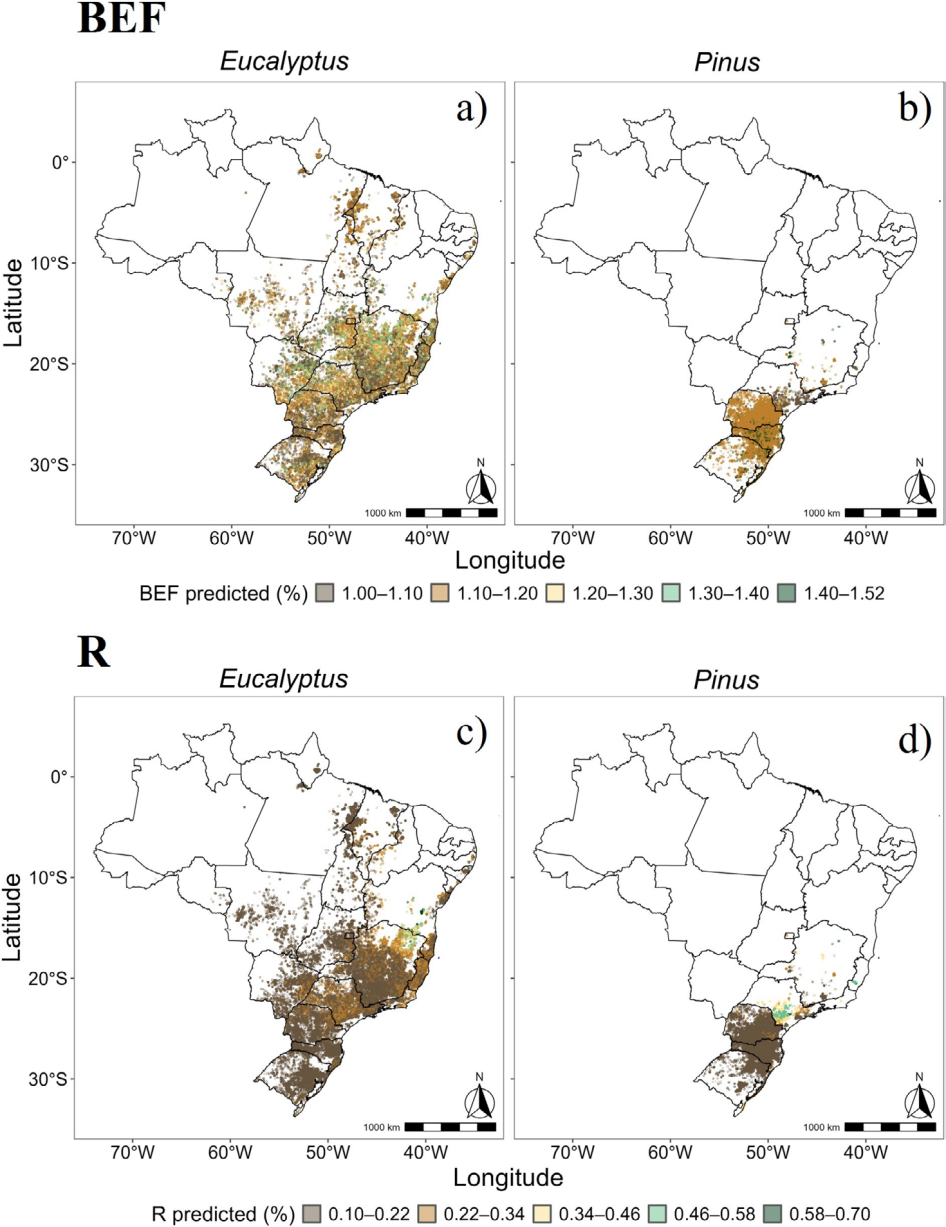
Biomass Expansion Factor (BEF) and Root‐to‐Shoot Ratio (*R*) estimates for commercial *Eucalyptus* and *Pinus* plantations. Panels a) and b) present the BEF estimates for *Eucalyptus* and *Pinus* plantations, respectively, while panels c) and d) display the R estimates for the same species, respectively. Map lines delineate study areas and do not necessarily depict accepted national boundaries.

**FIGURE 10:**
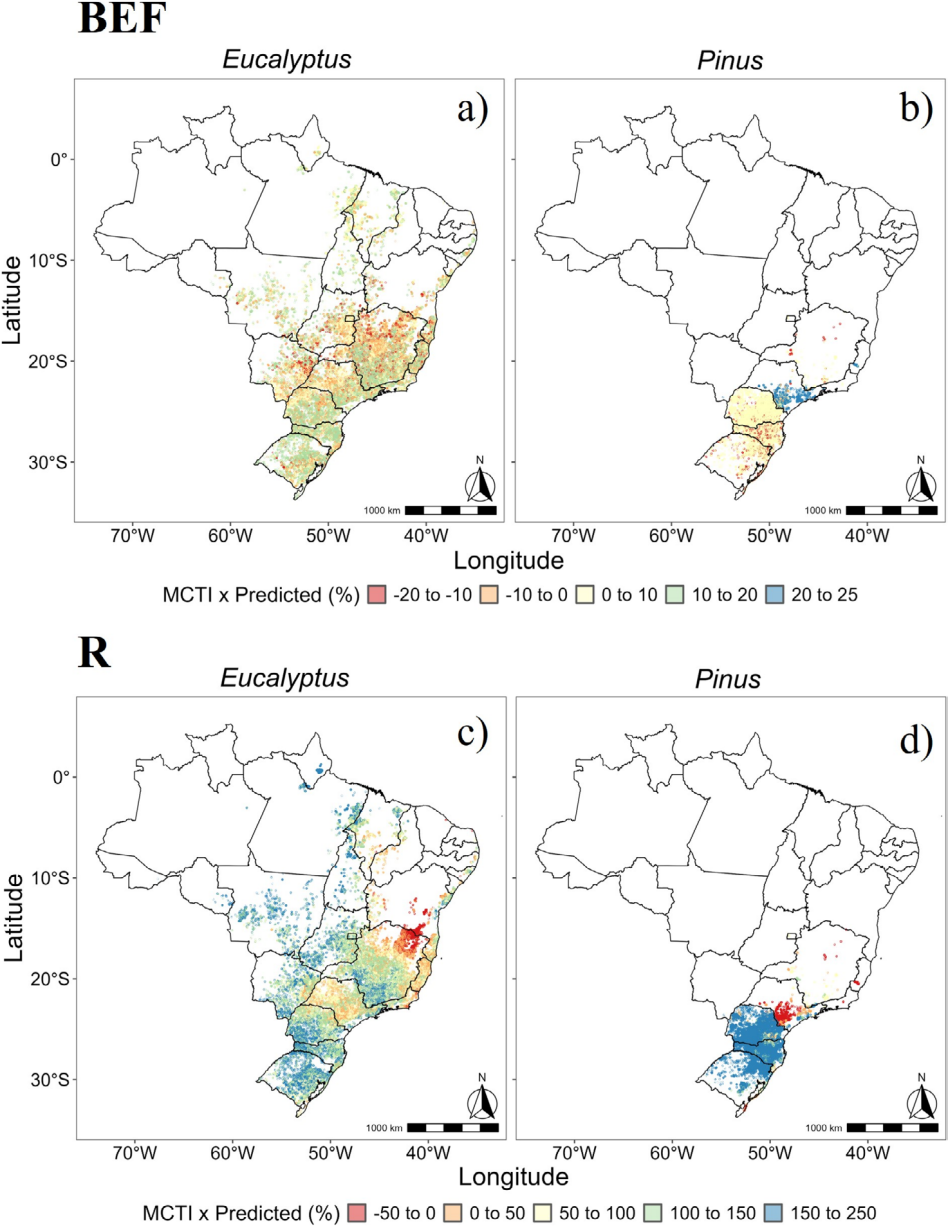
Benchmarking of Biomass Expansion Factor (BEF) and Root‐to‐Shoot Ratio (*R*) estimates from the study versus default MCTI values. Panels a) and b) present the percentage differences in BEF for *Eucalyptus* and *Pinus* plantations, respectively, while panels c) and d) display the percentage differences in R for the same species, respectively. Map lines delineate study areas and do not necessarily depict accepted national boundaries.

At the commercial scale, the estimated BEF for *Eucalyptus* averaged 1.15, ranging from 1.02 to 1.39 (Figure 9a), closely aligned with the experimental mean of 1.16. In contrast, the MCTI (2004) default value of 1.2 overestimated BEF by 4.9%. The states with the largest planted areas (Minas Gerais—MG, Mato Grosso do Sul—MS, and São Paulo—SP) showed differences of less than 5%, indicating strong agreement between our model predictions and the MCTI reference. However, larger deviations were observed in the Northern (7.1%) and Southern (6.4%) regions of the country (Figure 10a).

For *R* in *Eucalyptus*, the commercial‐scale mean was 0.20 (ranging from 0.1 to 0.7) (Figure 9c), 9.1% lower than the experimental mean (0.22) and 42.9% lower than the MCTI reference value (0.35). The percentage differences between the commercial scale and MCTI estimates varied widely, averaging 93.5% and ranging from 50% to 250%. In the Southeast, which has the largest planted area, the MCTI overestimated *R* by 78.9%, with Espírito Santo showing the lowest discrepancy (63.8%). The most pronounced overestimations were in the Northern (165.7%) and Southern (129.2%) regions (Figure 10c).

For *Pinus* spp., the estimated commercial‐scale BEF averaged 1.20 (ranging from 1.00 to 1.51) (Figure 9b), closely matching the experimental mean (1.22) with a difference of only 1.64%. However, the MCTI default value (1.25) systematically overestimated BEF by an average of 5.8%. In the main production region (Southern Brazil), the difference was below 3%, with Paraná showing the lowest discrepancy (1.68%). The most significant overestimation occurred in São Paulo (22.3%) (Figure 10b), a critical finding given the state's fourth‐place ranking in the total planted area (IBÁ 2024).

The commercial‐scale estimate for *R* in *Pinus* spp. was 0.18 (range: 0.1–0.7) (Figure 9d), substantially lower than the experimental mean (0.31). Applying the MCTI reference value (0.35) to commercial plantations resulted in an average deviation of 145.2%. The largest overestimations, exceeding 200%, were observed in Santa Catarina (SC) and Rio Grande do Sul (RS), the two states with the most extensive *Pinus* plantations (Figure 10d).

## 4 Discussion

### 4.1 Accuracy on Biomass Expansion Factors and Root‐To‐Shoot Ratios

Improving the accuracy of forest biomass and carbon estimates is crucial to ensuring that quality information is used for accounting purposes in compliance with UNFCCC and Paris Agreement commitments. Given the usefulness and readiness of biomass conversion and expansion factors (BCEFs) in converting tree measurements into biomass, choosing an appropriate BEF and *R* is fundamental to reducing uncertainties in forest biomass and carbon stock estimates. A notable issue lies in using fixed BEF and *R* or MCTI default values that often result in unrealistic estimates. A more robust approach involves applying regression models that capture variations in expansion/conversion factors based on dbh, tree height, and age to produce reliable estimates of forest tree biomass, carbon sinks, and CO_2_ equivalents (Sanquetta et al. 2011). In light of this knowledge gap, this study focused on developing models for BEF and *R* estimation for the two most common genera planted in Brazil, emphasizing changes in forest biomass, as a proxy for carbon stocks, under contrasting site conditions.

We modeled *Eucalyptus* and *Pinus* independently since species group (genera and forest type) or species‐specific BEF leads to more accurate aboveground biomass estimates at local and national scales (Kim et al. 2019; Ali et al. 2023). This approach is aligned with the statement that BEF varies with species (Luo et al. 2013), which was also evidenced by different BEF patterns in our study on *Eucalyptus* spp. and *Pinus* spp. In terms of the average BEF, that for *Pinus* spp. was 5.2% higher than that for *Eucalyptus* spp. This can be attributed to the fact that BEF is subject to variations depending on carbon allocation patterns (Lehtonen et al. 2004; Luo et al. 2013), with gymnosperms, due to their phylogeny, allocating more carbon to leaves than woody angiosperms (Poorter et al. 2012). This may explain why *Pinus* spp., compared with *Eucalyptus* spp., allocates a relatively higher proportion of aerial biomass to other aerial components than the stem. This finding corroborates the study by Campoe et al. (2020), who reported productivity allocation across six *Eucalyptus* clones, with an average foliage net primary production (FNPP) of 7% (5% up to 11%) and an average wood net primary production (WNPP) of 25% (16% up to 29%). Furthermore, the study by Deliberali (2015) showed that *Pinus caribaea* var. *hondurensis* had an average FNPP of 9% and a WNPP of 22%, while *Pinus taeda* presented an average FNPP of 11% and a WNPP of 9%.

The average BEF value for the *Eucalyptus* spp. found in this study (1.16) aligns with the average (1.17) observed by Hernández‐Ramos et al. (2017), who evaluated commercial plantations of *Eucalyptus urophylla* S. T. Blake aged between one and 7 years in Tabasco, Mexico. Both studies found values approximately 3% lower than the MCTI default (1.20). The average BEF for *Pinus* spp. in this study (1.22) was lower than the MCTI default (1.25) and the value found by Sanquetta et al. (2011) (1.47). However, it is worth pointing out that the differences can be attributed to the younger individuals included in their sample, beginning at 2 years of age, while in the present study, the youngest age was 7.3 years. Similar to previous studies involving other species (Jagodziński et al. 2019b; Teobaldelli et al. 2009), we found the same pattern where the youngest trees had the highest BEF values. This statement is supported by the moderate negative correlation between BEF and age, which was −0.67 in the study by Sanquetta et al. (2011) and −0.57 (Figure S3) in the present study. This negative linear association of BEF with age was also observed in the present study for *Eucalyptus* spp. at a stronger degree (Figure S3). This behavior indicates a decrease in the BEF value with increasing age since larger and older trees have lower proportions of leaves than younger and smaller ones (Sanquetta et al. 2011). The tendency of young trees to invest more mass into vegetative and reproductive organs per unit mass of supporting organs than old trees is a strategy to promote rapid growth in the early stages (Liang et al. 2024).

In this context, the differences in BEF were greater in younger trees than in older ones, regardless of genera. In alignment with our findings, similar behavior was observed by Jagodziński et al. (2019a) for a *Pinus sylvestris* L. chronosequence. These cited authors explained this major variability by the different growth conditions in the juvenile phase. Knowing the variations in BEF with aging, our BEF modeling incorporated age as a predictor among other variables, producing excellent estimates. The development of age‐dependent BEFs is encouraged to enhance the quality of carbon stock information, as the biomass allocation patterns change during the rotation, with stem wood proportion increasing with tree size. Therefore, age‐dependent BEFs are proper alternatives when no locally representative biomass equations are available (Petersson et al. 2012; Lehtonen et al. 2004).

This study also identified diameter and height as important drivers of BEF for *Eucalyptus* spp. For *Pinus* spp., these variables indirectly influenced the BEF variability, as they were incorporated into the model as the slenderness degree, defined as the ratio between height and diameter. Sanquetta et al. (2011) characterized the relationship between BEF with dbh, height, and age as a negative exponential curve, reinforcing these variables as important predictors of BEF for *Pinus* spp. in Brazil. The negative correlation between BEF and height for both genera in this study (Figure S3) is similar to that described in previous studies (Kim et al. 2019; Sanquetta et al. 2011). The decrease in BEF with increasing height was related to a greater proportion of trunk biomass, whereas the other biomass components of the tree declined or showed less change (Kim et al. 2019). This may be because diameter and height are among the variables that are highly correlated with biomass production (Jagodziński et al. 2019b) and the allocation strategies of trees (Lehtonen et al. 2004).

Climate is also a source of variation in forest biomass, which is highly important for accurate estimates of carbon accounting (He et al. 2021). In this sense, the use of climate in BEF models is crucial in reducing uncertainties. Luo et al. (2013) identified different BEF–climate relationships depending on forest type. In our study, temperature and precipitation were weakly correlated with BEF and exhibited different trends according to genus (Figure S3). This weak linear association in the case of *Eucalyptus* spp. can be attributed to the fact that our database included clones with distinct acclimatization patterns, as demonstrated in the study by Queiroz et al. (2020) that *Eucalyptus* genotypes differ in terms of thermal demands for growth.

In our study, the thermal influence on BEF was best expressed as a cause‐and‐effect relationship by incorporating temperature classes, which emerged as a significant predictor to explain variations observed in *Eucalyptus* spp. Binkley et al. (2017), when analyzing clonal *Eucalyptus* plantations along a climatic gradient from Brazil to Uruguay, revealed that the effect of temperature was stronger than that of precipitation. Temperature increases lead to a decline in stem wood production. Campoe et al. (2020) confirmed that the most stressful sites showed greater allocation of foliage relative to stem production compared to intermediate and moderate sites. Furthermore, temperature was more strongly related to carbon flux and partitioning than water stress for *Eucalyptus* plantations, with carbon partitioning shifting from aboveground to belowground with increasing temperature.

The relationship between belowground and aboveground biomass, described by the root‐to‐shoot ratio (*R*), is driven by several factors, with one general trend being its increase under drier conditions (Barton and Montagu 2006). This statement corroborates our finding, which showed the highest root‐to‐shoot ratio for the most stressful sites for both genera. Barton and Montagu (2006) demonstrated a strong influence of water availability on the root‐to‐shoot ratio of *Eucalyptus camaldulensis* , with the ratio doubling under conditions of reduced water availability. In line with these findings, precipitation was negatively correlated with *R* and also acted as a significant predictor that improved the accuracy of estimates for both genera. This decreasing trend is explained by the fact that increased precipitation contributes to a sufficient supply of water, which causes trees to allocate less biomass to their roots to absorb moisture from the soil (Luo et al. 2012).

We also highlight diameter as a driving factor of *R* behavior due to its significant effect on *Eucalyptus* spp. estimates of *R*. This is supported by Barton and Montagu (2006), who demonstrated that the relationship between diameter and belowground biomass varies greatly in *Eucalyptus camaldulensis* , with an increase in *R* as trees become larger. The authors also revealed that water availability drives this relationship, being a significant factor in the amount of coarse roots for a given dbh. Greater water availability implies a lower production of coarse root biomass and, consequently, a reduction in *R*. Regarding *Pinus* spp., we identified total height as a key factor closely associated with *R*, revealing a decreasing trend and serving as a significant predictor. A similar trend was observed by Sanquetta et al. (2011) when analyzing *Pinus elliottii* and *Pinus taeda* in southern Brazil, indicating that the use of height enhances estimates of *R*. In predictive terms, age captured variations in *R* for both genera. This behavior is attributed to older trees having proportionally less root biomass than younger trees (Sanquetta et al. 2011). Furthermore, the cited authors reported that as *Pinus elliottii* and *Pinus taeda* grow and advance in age, the investment in foliage and roots to total biomass declines. The present study supports this finding since a clear declining trend in *R* was shown with increasing age for *Pinus* spp. Our analysis resulted in an average *R* of 0.31 for *Pinus* spp., approximately 11.4% lower than the MCTI (2004) default (0.35) and 45.2% higher than that found by Sanquetta et al. (2011). The contrasting results between our study and the study of Sanquetta et al. (2011) can be attributed to the different species types and the younger ages sampled in their study.

The extensive data set used in this study consists of a massive nationwide sampling effort that surpasses previous studies at the national scale. It covers a wide range of tree growth stages and climatic conditions in contrasting regions of Brazil. Given the reliability of both the representativeness and consistency of the dataset for the Brazilian forestry sector, we successfully identified the sources of variation affecting the BEF and *R* estimates. These sources of variation at the tree level depend on the response variable (BEF or *R*) and the genus and include diameter, total height, age or age class, Köppen climate classification, species, slenderness degree, rainfall, and temperature class.

The use of temperature classes to estimate BEF for *Eucalyptus* spp. resulted in errors of less than 1%, while the use of the MCTI (2004) default value led to an overestimation of local stocks of up to 12.9%. For *Pinus* spp., the age class partially captured the variation in BEF, with errors of less than 0.5%, in contrast to the MCTI default value, which resulted in underestimating the aboveground stock of up to 14%. The *R* estimates in this study exhibited larger errors for the younger age classes, generally underestimating the belowground carbon stock by less than 4%. In contrast, the MCTI default overestimated the stock by 70.8% and 61.3% for the youngest ages of *Eucalyptus* spp. and the oldest age classes of *Pinus* spp., respectively. In light of these findings, it is important to emphasize that the use of fixed values, whether MCTI defaults or our averages, led to inaccurate estimates. Therefore, we reinforce the recommendation of Sanquetta et al. (2011), highlighting that the use of regression equations produces better estimates. This approach ensures more reliable carbon accounting in regions with diverse climates and growing conditions.

### 4.2 Applicability and Global Implications

The BEF and *R* models presented in this study address a critical gap in biomass and carbon estimation, particularly in the absence of volume and biomass equations for specific stands. This contribution is especially relevant given the limited number of studies on BEF and *R*, which are often regionally restricted and based on small sample sizes. Therefore, compared to the existing literature for BEF and *R* estimation, the strengths of this study lie in: (i) the BEF and *R* equations are based on the allometry of trees from the regionally representative database, and (ii) our database serves as a key resource for large‐scale carbon accounting, encompassing extensive nationwide data that captures diverse tree growth stages and a wide range of edaphoclimatic conditions. This study also incorporates a rigorous data consistency analysis to ensure reliability throughout the modeling process, thereby enabling the development of accurate and robust models.

Good practice in adopting these species‐specific BEF and *R* models is crucial to proposing realistic carbon sequestration projects. It is essential to acknowledge that for *Pinus* spp., the application of the BEF and *R* models is restricted to *Pinus caribaea var. hondurensis* (PCH) and *Pinus taeda* (PTA), with the latter being the most representative species in the sample set used in this study. Notably, species‐specific BEF models should only be applied to stands within specific age ranges: 2–8.4 years for *Eucalyptus* spp. and 7.3–17.3 years for *Pinus* spp. For *Eucalyptus* spp., particular caution is advised when applying the BEF model to young stands, especially those under 3 years. Residual analyses indicated larger deviations in this early growth stage, reflecting reduced model accuracy. These deviations are attributed to the relatively high observed BEF values at younger ages, which the model struggles to capture accurately. Furthermore, to guarantee the reliability of BEF and *R* predictions, it is essential to ensure that estimations are made within the appropriate diameter and height ranges. Specifically, BEF predictions are applicable within dbh: 3.21–28.2 cm and ht.: 6.0–35.7 m for *Eucalyptus* spp., and dbh: 9.99–34.7 cm and ht.: 8.8–27.0 m for *Pinus* spp. Similarly, *R* estimations should be considered within dbh: 7.0–26.6 cm and ht.: 10.6–34.1 m for *Eucalyptus* spp., and dbh: 12.99–29.5 cm and ht.: 8.8–24.95 m for *Pinus* spp.

In view of these considerations, the species‐specific BEF and *R* models developed in this study are suitable for broad‐scale applications in the national context and other regions with similar climate and growth conditions. These estimates provide essential support for Brazil and other countries in producing high‐quality greenhouse gas (GHG) inventories and in guiding efforts to achieve Nationally Determined Contributions (NDCs) and the goals of the Paris Agreement for forest plantations. Furthermore, the methodological approach and predictor variables selected in this study can be replicated to develop BEF and *R* models based on reliable data for areas with different species composition and environmental conditions, extending their applicability to a wider range of contexts. A key consideration is that these models were originally developed using individual tree data from forests without thinning interventions; consequently, the dbh range in our dataset is more restricted, reflecting the stand dynamics typical of forests without thinning.

## 5 Conclusion

We compiled an extensive dataset that covers a wide range of tree growth stages and climatic conditions, being the most representative of BEF and *R* sampling for the forest sector in Brazil. Our study fills a gap in the knowledge of BEF and *R* patterns for *Eucalyptus* spp. and *Pinus* spp. and advances our understanding of their driving factors.

The findings suggest that BEF and *R* functions, including their controlling factors, are essential for reducing uncertainty when estimating forest biomass and carbon stocks at local and national scales. These models can be used in other regions under similar conditions. In the BEF prediction, the random effects in the linear mixed model that contributed significantly to capturing the variations in *Eucalyptus* spp. and *Pinus* spp. were the temperature and age classes, respectively. *R* estimates were primarily influenced by precipitation and age for both genera, with the slenderness degree and diameter specifically affecting *Eucalyptus* spp., whereas height was a key factor for *Pinus* spp.

For these reasons, fixed or default values for BEF and *R* should be discouraged in locations with different climates and growing conditions, avoiding biased estimates for carbon accounting and greenhouse gas inventories. To further advance the current state of the art of BEF and *R*, we suggest modeling focused on climate variables to deepen our knowledge of their spatial patterns facing future climate change.

## Author Contributions

**Isáira Leite e Lopes:** conceptualization, data curation, formal analysis, investigation, methodology, software, visualization, writing – original draft, writing – review and editing. **Otávio Camargo Campoe:** conceptualization, data curation, formal analysis, funding acquisition, investigation, methodology, project administration, resources, visualization, writing – original draft, writing – review and editing. **Geovanni Malatesta Barros:** conceptualization, data curation, formal analysis, investigation, methodology, writing – original draft, writing – review and editing. **Anny Francielly Ataide Gonçalves:** conceptualization, data curation, formal analysis, investigation, methodology, writing – review and editing. **Gabriela Gonçalves Moreira Matzner:** data curation, investigation, writing – review and editing. **Marco Aurelio Figura:** conceptualization, data curation, formal analysis, investigation, methodology, writing – review and editing. **Clayton Alcarde Alvares:** conceptualization, data curation, formal analysis, investigation, methodology, validation, visualization, writing – review and editing. **Fernanda Leite Cunha:** conceptualization, data curation, formal analysis, investigation, methodology, writing – review and editing. **Verônica Boarini Sampaio de Rezende:** conceptualization, investigation, methodology, supervision, writing – review and editing. **James Stahl:** data curation, writing – review and editing. **Josiana Jussara Nazaré Basílio:** conceptualization, data curation, formal analysis, investigation, writing – review and editing. **Hyngrid Jaiely Araújo Félix:** investigation, visualization, writing – review and editing. **Thiago Bortoluzzi Boigues:** data curation, writing – review and editing. **Grasiele Dick:** writing – review and editing. **Eduardo Moré de Mattos:** writing – review and editing. **Joannès Guillemot:** investigation, writing – review and editing. **Guerric Le Maire:** investigation, writing – review and editing. **Rachel Cook:** investigation, writing – review and editing. **Timothy J. Albaugh:** investigation, writing – review and editing. **Rafael Alejandro Rubilar:** investigation, writing – review and editing. **Márcia Silva de Jesus:** writing – review and editing. **Adriano Scarpa Tonaco:** writing – review and editing. **Juliana Soares Biruel Munhoz:** data curation, investigation. **Isabel Deliberari:** data curation, investigation. **Jose Luiz Stape:** investigation, writing – review and editing. **Yann Nouvellon:** investigation, writing – review and editing. **Jean‐Paul Laclau:** investigation, writing – review and editing.

## Conflicts of Interest

The authors declare no conflicts of interest.

## Supporting information


**Data S1:** gcb70395‐sup‐0001‐Supinfo.docx.

## Data Availability

The data that support the findings of this study are openly available in DRYAD at https://doi.org/10.5061/dryad.5x69p8dhk.
