# Supplementary material for: Improved Estimates of Biomass Expansion Factors and Root‐To‐Shoot Ratios: An Approach for Different Forest Types Across a Climatic Gradient in Brazil
Source: Glob Chang Biol. 2025 Aug 4;31(8):e70395. doi: 10.1111/gcb.70395 (PMC12319663; doi:10.1111/gcb.70395)
Supplement: Supplementary file 1 — Data S1: gcb70395‐sup‐0001‐Supinfo.docx. [file GCB-31-e70395-s001.docx]

# **Supplementary material**

#
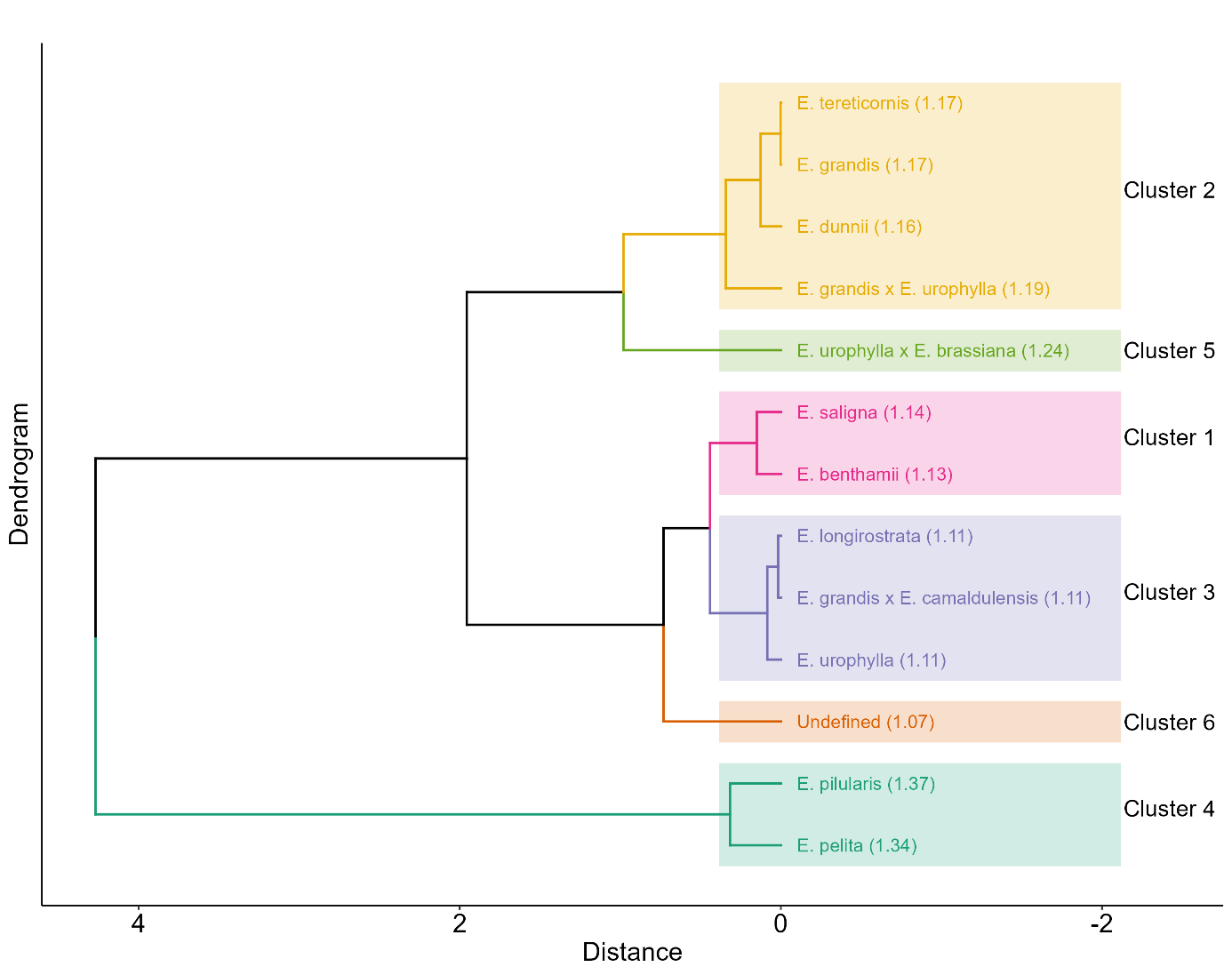


Figure S1. Species grouping according to the biomass expansion factor (BEF) for *Eucalyptus*


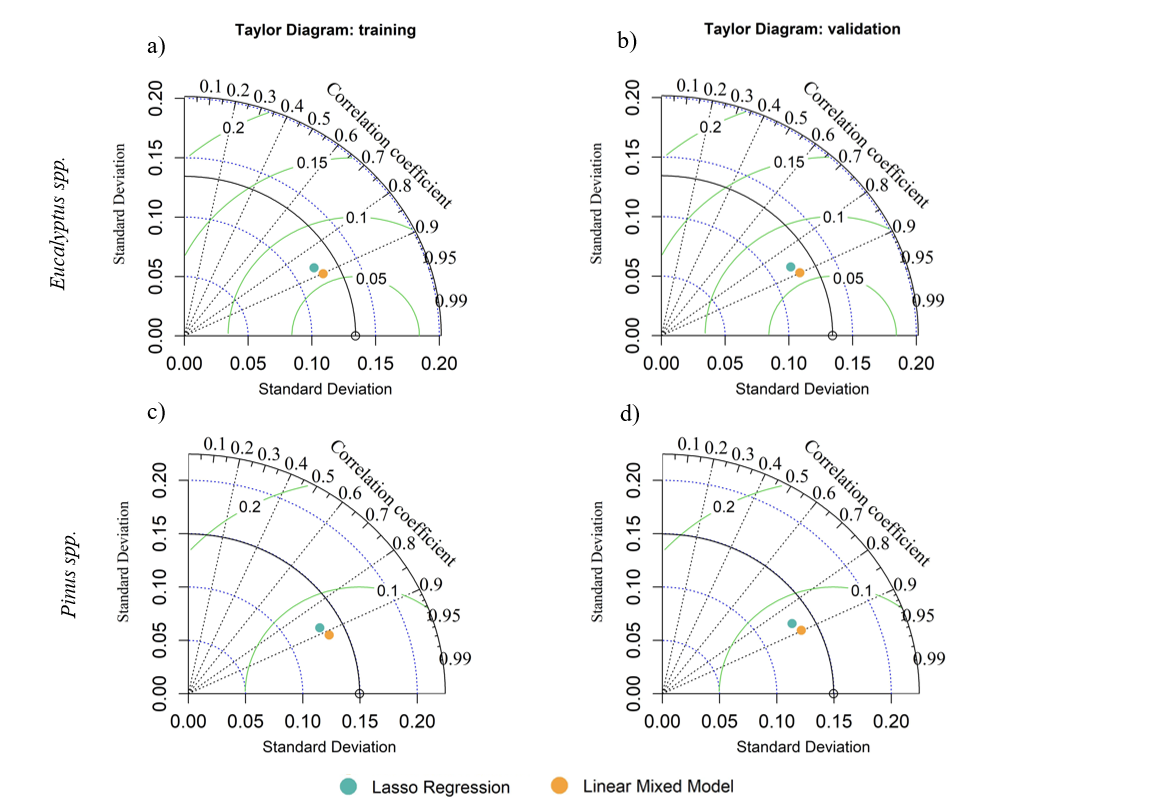


Figure S2. Performance evaluation of biomass expansion factor (BEF) modeling methods using the Taylor diagram.


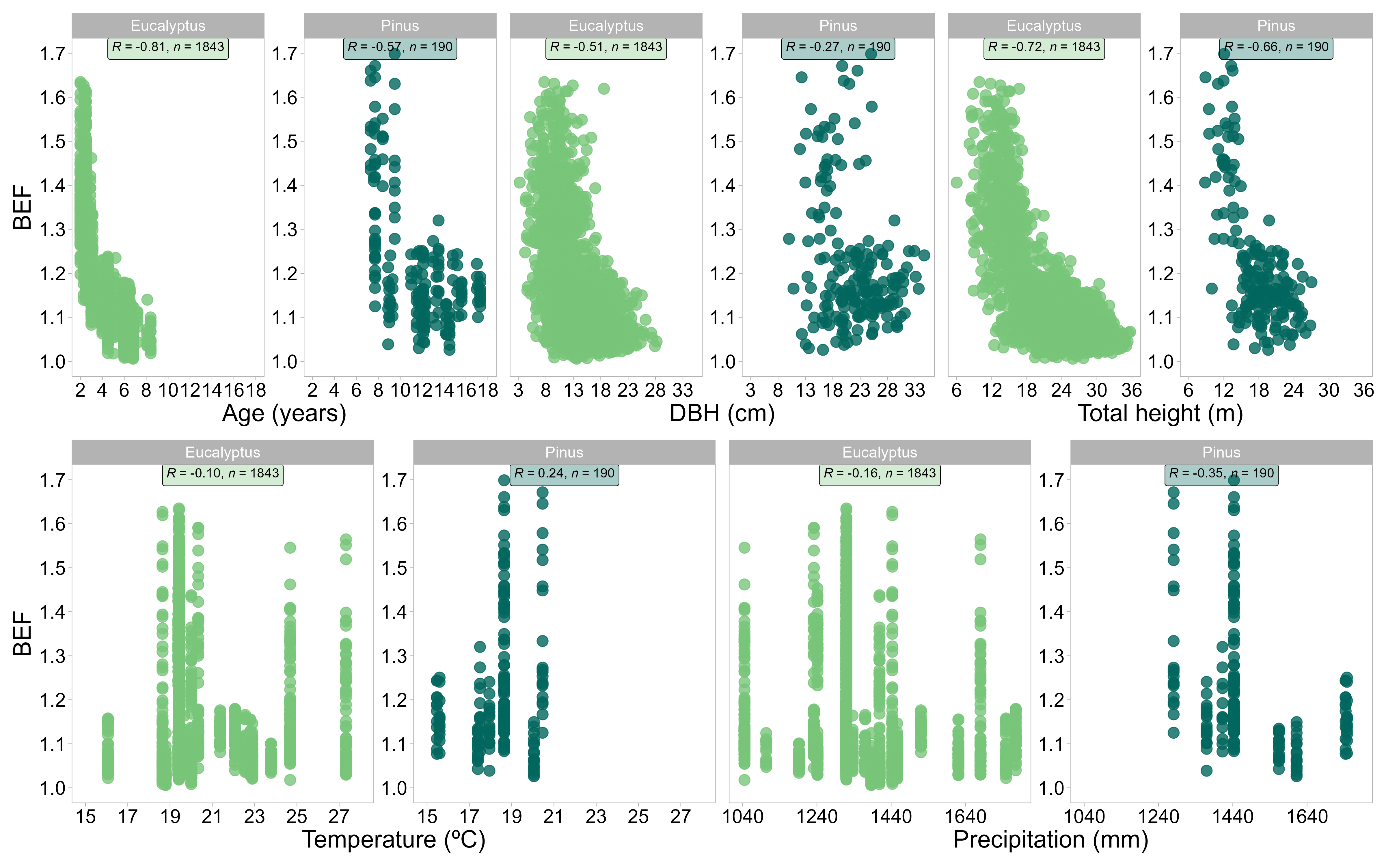


Figure S3. Scatterplots and correlation values for the biomass expansion factor (BEF) for each genus.


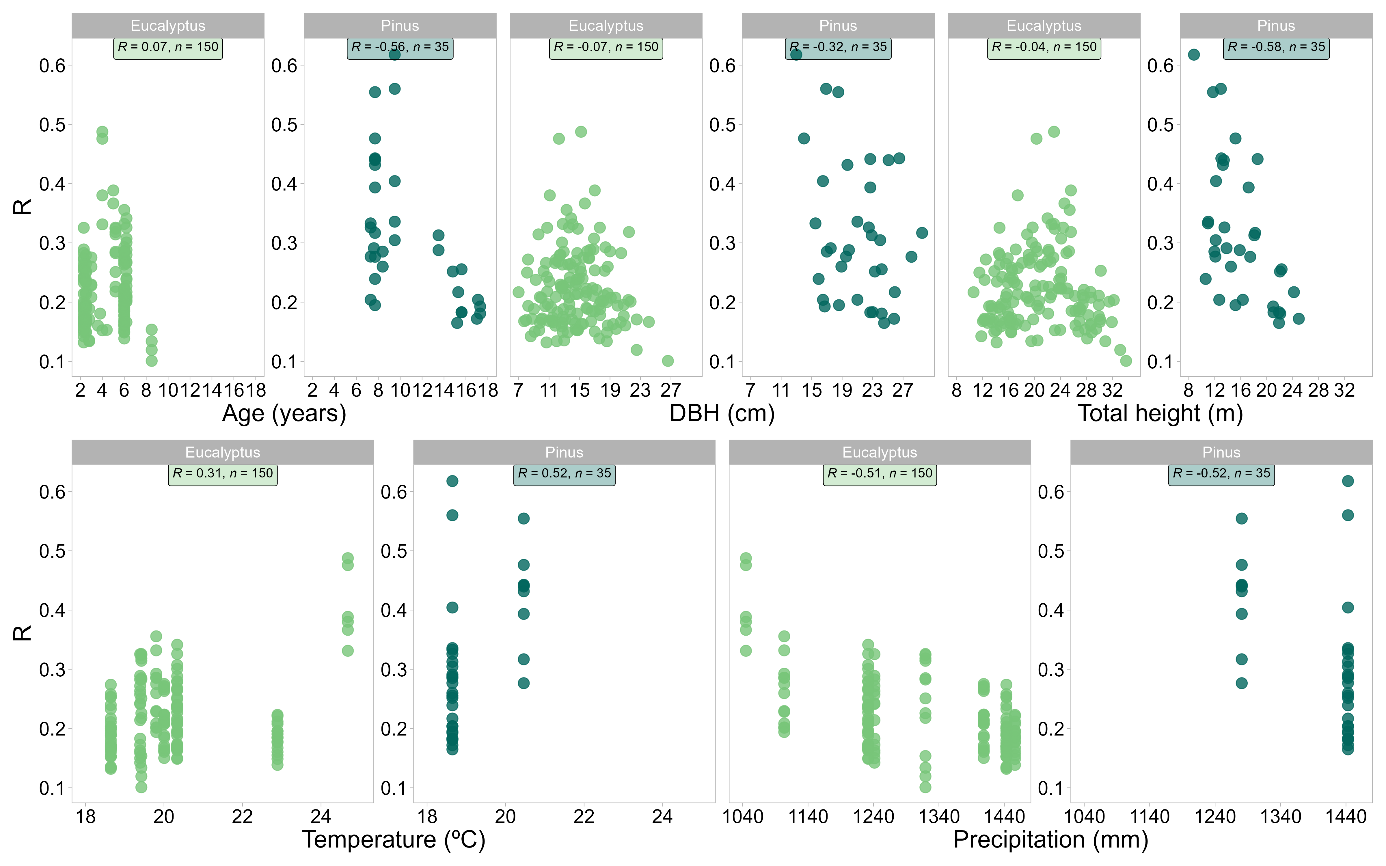


Figure S4. Scatterplots and correlation values for R for each genus.


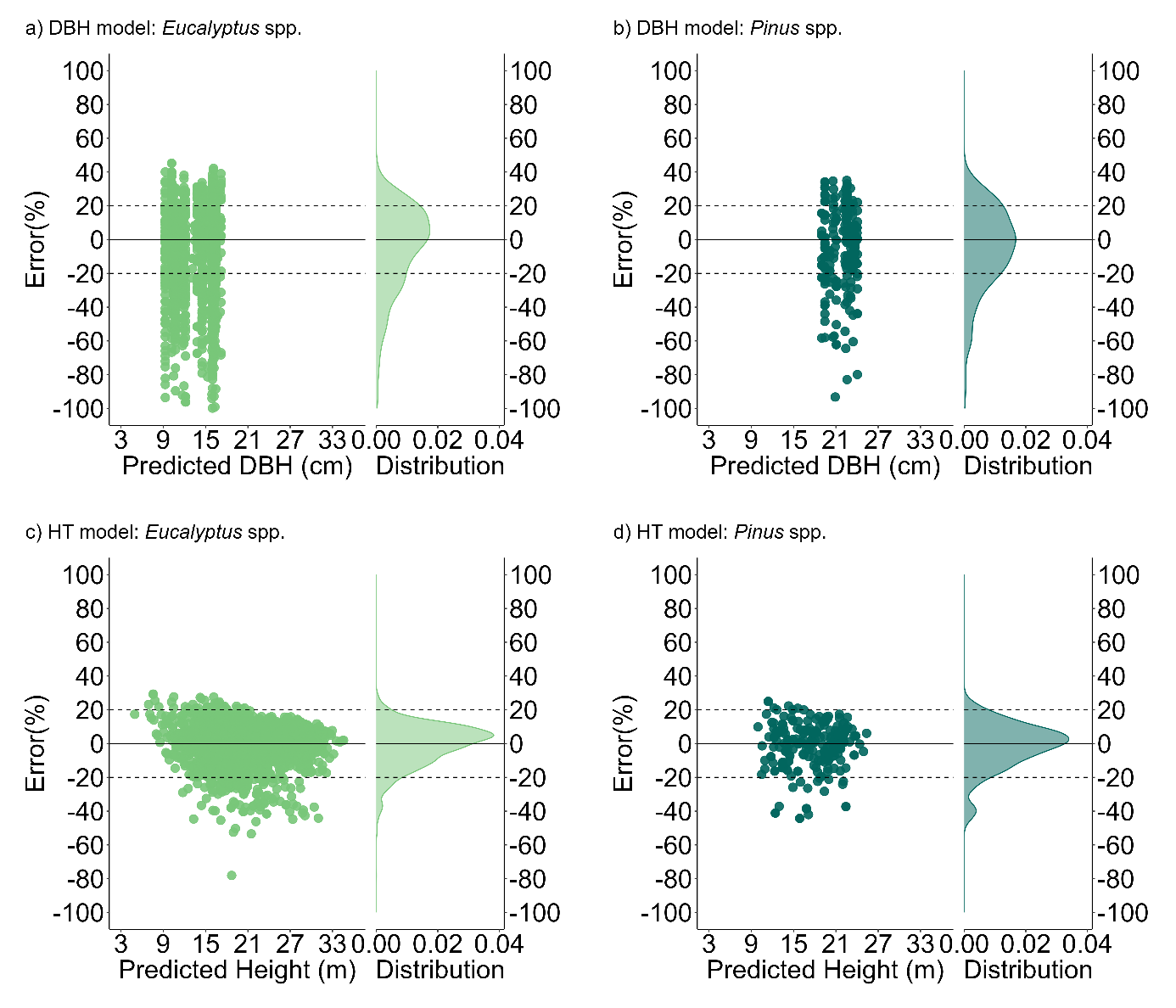


Figure S5. Residual plots and marginal histograms of dbh (cm) and total height (m) predictions.

Table S1. Site characteristics and number of biomass expansion factor (BEF) and root-to-shoot ratio (R) samples for the genera *Eucalyptus* and *Pinus*.

| BEF – *Eucalyptus* spp. | | | | | | | | |
| --- | --- | --- | --- | --- | --- | --- | --- | --- |
| Site | Lat. | Long. | Nearest city | Köppen | n | Tmed | Rmed | Age |
| 1 | -11.86 | -38.37 | Inhambupe | As | 109 | 24,68 | 1045,12 | 2–5.9 |
| 2 | -16.34 | -39.6 | Eunápolis | Aw | 42 | 23,77 | 1191,87 | 6.2 |
| 3 | -24.2 | -50.5 | Telêmaco Borba | Cfb | 7 | 18,65 | 1443,11 | 5.9 |
| 4 | -24.23 | -50.53 | Telêmaco Borba | Cfb | 92 | 18,65 | 1443,11 | 2.3–6.2 |
| 5 | -22.35 | -46.97 | Mogi Guaçu | Cwa | 141 | 20,01 | 1408,5 | 2.4–6 |
| 6 | -23.85 | -48.7 | Buri | Cfa | 104 | 19,39 | 1241,51 | 2.3–6.4 |
| 7 | -18.02 | -50.9 | Rio Verde | Aw | 37 | 22,07 | 1775,53 | 6 |
| 8 | -19.96 | -51.59 | Inocência | Am | 48 | 22,8 | 1621,09 | 6.3 |
| 9 | -3.44 | -43.07 | Santa Quitéria do Maranhão | As | 74 | 27,32 | 1680,48 | 2.3–6.5 |
| 10 | -23.1 | -48.6 | Itatinga | Cfa | 861 | 19,42 | 1319,42 | 2–8.4 |
| 11 | -27.53 | -50.1 | Otacílio Costa | Cfb | 34 | 16,07 | 1749,2 | 6.4 |
| 12 | -20.9 | -51.9 | Três Lagoas | Aw | 56 | 22,89 | 1456,19 | 6 |
| 13 | -24.21 | -49.97 | Arapoti | Cfb | 49 | 18,79 | 1386,35 | 6.8 |
| 14 | -18.73 | -47.92 | Araguari | Aw | 28 | 21,37 | 1520,08 | 6.4 |
| 15 | -19.31 | -42.42 | Belo Oriente | Aw | 33 | 22,56 | 1370,16 | 6.5 |
| 16 | -23.03 | -48.53 | Botucatu | Cfa | 42 | 19,68 | 1336,11 | 6.4 |
| 17 | -17.32 | -43.77 | Olhos-d'Água | Cwa | 74 | 20,34 | 1231,92 | 2.5–6.2 |
| 18 | -17.69 | -42.52 | Capelinha | Cwb | 12 | 19,8 | 1103,65 | 3–6 |
| R – *Eucalyptus* spp. | | | | | | | | |
| 1 | -11.86 | -38.37 | Inhambupe | As | 6 | 24,68 | 1045,12 | 4–5 |
| 4 | -24.23 | -50.53 | Telêmaco Borba | Cfb | 30 | 18,65 | 1443,11 | 2.3–6.2 |
| 5 | -22.35 | -46.97 | Mogi Guaçu | Cwa | 20 | 20,01 | 1408,5 | 6 |
| 6 | -23.85 | -48.7 | Buri | Cfa | 15 | 19,39 | 1241,51 | 2.3 |
| 10 | -23.1 | -48.6 | Itatinga | Cfa | 13 | 19,42 | 1319,42 | 5.2–8.5 |
| 12 | -20.9 | -51.9 | Três Lagoas | Aw | 16 | 22,89 | 1456,19 | 6 |
| 17 | -17.32 | -43.77 | Olhos-d'Água | Cwa | 37 | 20,34 | 1231,92 | 2.5–6.2 |
| 18 | -17.69 | -42.52 | Capelinha | Cwb | 13 | 19,8 | 1103,65 | 3–6 |
| BEF – *Pinus* spp. | | | | | | | | |
| 1 | -19.22 | -47.73 | Nova Ponte | Cwb | 16 | 20,05 | 1611,82 | 11.7–14.5 |
| 2 | -22.69 | -47.65 | Piracicaba | Cfa | 20 | 20,46 | 1280,71 | 7.7 |
| 3 | -24.09 | -49.49 | Sengés | Cfb | 18 | 17,95 | 1369,54 | 8.9–9.3 |
| 4 | -24.23 | -50.53 | Telêmaco Borba | Cfb | 69 | 18,65 | 1443,11 | 7.3–17.3 |
| 5 | -24.41 | -50.49 | Telêmaco Borba | Cfb | 15 | 18,65 | 1443,11 | 12–12.3 |
| 6 | -24.36 | -49.79 | Jaguariaíva | Cfb | 12 | 17,5 | 1412,19 | 13–13.5 |
| 7 | -26.04 | -50.25 | Três Barras | Cfb | 16 | 17,4 | 1564,15 | 12.2–14.2 |
| 8 | -26.75 | -50.73 | Lebon Régis | Cfb | 12 | 15,47 | 1743,8 | 11–11.5 |
| 9 | -27.16 | -50.56 | Ponte Alta do Norte | Cfb | 12 | 15,59 | 1747,33 | 11.7–12 |
| R – *Pinus* spp. | | | | | | | | |
| 2 | -22.69 | -47.65 | Piracicaba | Cfa | 9 | 20,46 | 1280,71 | 7.7 |
| 4 | -24.23 | -50.53 | Telêmaco Borba | Cfb | 26 | 18,65 | 1443,11 | 7.3–17.3 |

Table S2. Random-effects parameters of linear mixed models used to predict BEF for *Eucalyptus* and *Pinus* plantations.

| Random effects | | |
| --- | --- | --- |
| *Eucalyptus* spp. | | |
| Temperature class (ºC) | Intercept (*b*_0j_) | Slope (*b*_1j_) |
| (16.0-18.0] | -0.87797 | 0.036906 |
| (18.0-18.9] | -0.61262 | 0.025684 |
| (18.9-19.8] | -0.67874 | 0.028709 |
| (19.8-20.8] | -0.63558 | 0.026105 |
| (20.8-21.7] | -0.82192 | 0.036107 |
| (21.7-22.6] | -0.64949 | 0.028747 |
| (22.6-23.6] | -0.56626 | 0.024561 |
| (23.6-24.5] | -0.62089 | 0.026162 |
| (24.5-25.4] | -0.76925 | 0.03211 |
| (25.4-27.3] | -0.93966 | 0.039706 |
| *Pinus* spp. | | |
| Age class (Years) | Intercept (*b*_0j_) | Slope (*b*_1j_) |
| (7.3, 8.3] | 0.268521432 | - |
| (8.3, 9.3] | 0.277221163 | - |
| (9.3, 10.3] | 0.204682745 | - |
| (10.3, 11.3] | -0.062979795 | - |
| (11.3, 12.3] | -0.11622278 | - |
| (12.3, 13.3] | -0.105844512 | - |
| (13.3, 14.3] | -0.111757392 | - |
| (14.3, 15.3] | -0.111729372 | - |
| (15.3, 16.3] | -0.121310671 | - |
| (16.3, 17.3] | -0.120580817 | - |

Tabela S3. Adjusted models for predicting height and diameter in commercial-scale *Eucalyptus* and *Pinus* plantations as a function of age.

| Models form | Forest type | | A | b | R² | | RMSE  (%) | MAE |
| --- | --- | --- | --- | --- | --- | --- | --- | --- |
| $DBH=A(1-{exp}^{-b.Age})$ | Eucalyptus | | 18.04550 | 0.36156 | 0.38 | | 22.7 | 2.6 |
| $DBH=A(1-{exp}^{-b.Age})$ | Pinus | | 24.85697 | 0.19783 | 0.12 | | 21.6 | 3.8 |
| Models form | Forest type | β_0_ | β_1_ | β_2_ | R² | RMSE  (%) | | MAE |
| $\sqrt{HT}=\beta_{0}+\beta_{1}\ln DBH+\beta_{2}AC$ | Eucalyptus | 0.86930 | 1.50040 | -0.39213 | 0.84 | 11.2 | | 1.9 |
| $HT=\beta_{0}+\beta_{1}ln DBH+\beta_{2}Age$ | Pinus | -14.17204 | 7.17775 | 0.86171 | 0.75 | 12.0 | | 1.7 |

A, b, and β_i_ are the estimated parameters of the models. All parameters were statistically significant at the 5% significance level. Models met the assumption of homogeneity of variance (Breusch-Pagan test). AC is a dummy variable representing age class: 1 for trees aged ≤5 years and 0 for trees aged >5 years.

Table S4. Biomass expansion factor (BEF) and root-to-shoot ratio (R) for Eucalyptus and Pinus plantations by states at commercial scale.

| *Eucalyptus* genera | | | | | | | |
| --- | --- | --- | --- | --- | --- | --- | --- |
| State | Age range | T | P | BEF mean (range) | BEF dif (%) | R mean  (range) | R dif (%) |
| AC | 6.1 | 24.8 | 2149.6 | 1.07 | 12.5 | 0.14 | 158.8 |
| AL | 2.1-8.4 | 25.2 | 1561.8 | 1.12 (1.04-1.36) | 7.6 | 0.17 (0.11-0.56) | 111.7 |
| AM | 2.1-7.4 | 27.2 | 2137.4 | 1.16 (1.08-1.24) | 3.6 | 0.12 (0.11-0.17) | 193.3 |
| AP | 2.1-8.4 | 27.2 | 2957.6 | 1.12 (1.08-1.24) | 7.6 | 0.11 (0.10-0.15) | 213.7 |
| BA | 2.1-8.4 | 24.0 | 1352.7 | 1.18 (1.02-1.39) | 2.3 | 0.23 (0.10-0.70) | 70.0 |
| CE | 3.4-7.4 | 26.7 | 1009.5 | 1.11 (1.08-1.14) | 7.8 | 0.33 (0.26-0.40) | 10.2 |
| DF | 2.4-8.1 | 21.0 | 1531.5 | 1.14 (1.10-1.27) | 5.1 | 0.17 (0.14-0.20) | 109.6 |
| ES | 2.1-8.4 | 21.8 | 1284.5 | 1.16 (1.02-1.39) | 4.5 | 0.22 (0.15-0.38) | 63.8 |
| GO | 2.1-8.4 | 22.0 | 1640.8 | 1.17 (1.04-1.39) | 3.2 | 0.17 (0.11-0.26) | 120.8 |
| MA | 2.1-8.4 | 27.0 | 1546.7 | 1.14 (1.06-1.24) | 5.9 | 0.18 (0.11-0.36) | 104.8 |
| MG | 2.1-8.4 | 20.1 | 1368.6 | 1.15 (1.02-1.39) | 4.6 | 0.22 (0.11-0.66) | 77.5 |
| MS | 2.1-8.4 | 22.7 | 1534.4 | 1.17 (1.04-1.39) | 3.1 | 0.18 (0.11-0.27) | 102.2 |
| MT | 2.1-8.4 | 24.7 | 1996.3 | 1.15 (1.04-1.39) | 5.0 | 0.14 (0.10-0.27) | 156.4 |
| PA | 2.1-8.4 | 27.2 | 2191.4 | 1.12 (1.08-1.24) | 7.5 | 0.14 (0.10-0.20) | 165.2 |
| PB | 2.1-7.4 | 25.6 | 1497.1 | 1.14 (1.06-1.24) | 5.8 | 0.16 (0.15-0.22) | 115.2 |
| PE | 2.9-8.1 | 25.0 | 1296.0 | 1.10 (1.05-1.24) | 9.7 | 0.25 (0.12-0.41) | 50.8 |
| PI | 2.1-8.4 | 26.3 | 1274.5 | 1.14 (1.05-1.30) | 5.8 | 0.22 (0.13-0.38) | 69.4 |
| PR | 2.1-8.4 | 19.0 | 1538.5 | 1.16 (1.02-1.37) | 4.3 | 0.18 (0.10-0.31) | 107.4 |
| RJ | 2.1-8.4 | 20.1 | 1308.2 | 1.14 (1.02-1.39) | 5.7 | 0.22 (0.12-0.43) | 71.5 |
| RN | 2.1-4.4 | 25.9 | 1199.8 | 1.23 (1.11-1.24) | -2.1 | 0.24 (0.18-0.62) | 70.0 |
| RO | 2.1-8.4 | 25.5 | 2411.8 | 1.13 (1.05-1.26) | 6.5 | 0.11 (0.10-0.16) | 215.7 |
| RS | 2.1-8.4 | 17.9 | 1784.3 | 1.13 (1.03-1.36) | 7.0 | 0.15 (0.10-0.30) | 141.9 |
| SC | 2.1-8.4 | 18.1 | 1720.3 | 1.12 (1.02-1.36) | 7.8 | 0.15 (0.10-0.26) | 131.4 |
| SE | 2.1-8.4 | 25.6 | 1346.3 | 1.12 (1.05-1.30) | 7.5 | 0.21 (0.13-0.33) | 72.8 |
| SP | 2.1-8.4 | 19.2 | 1437.8 | 1.15 (1.02-1.37) | 4.9 | 0.20 (0.10-0.34) | 86.6 |
| TO | 2.1-8.4 | 26.6 | 1715.5 | 1.13 (1.05-1.32) | 6.3 | 0.15 (0.10-0.26) | 138.2 |
| *Pinus* genera | | | | | | | |
| State | Age range | T | P | BEF mean (range) | BEF dif (%) | R mean  (range) | R dif (%) |
| ES | 7.6-10.6 | 18.7 | 1249.9 | 1.01 (1.00-1.14) | 24.4 | 0.52 (0.50-0.64) | -33.10 |
| GO | 10.6 | 21.0 | 1531.5 | 1.15 | 9.0 | 0.23 | 50.4 |
| MG | 7.4-10.6 | 17.4 | 1568.8 | 1.18 (1.00-1.47) | 7.1 | 0.21 (0.11-0.70) | 73.2 |
| MS | 10.6 | 21.6 | 1707.6 | 1.00 | 25.0 | 0.14 | 150.0 |
| PR | 7.4-17.1 | 17.6 | 1600.5 | 1.24 (1.14-1.51) | 1.7 | 0.18 (0.10-0.52) | 126.5 |
| RJ | 10.6 | 17.2 | 1383.4 | 1.15 | 9.0 | 0.38 (0.21-0.45) | 5.0 |
| RS | 7.4-17.1 | 16.4 | 1804.1 | 1.20 (1.14-1.51) | 4.6 | 0.13 (0.10-0.45) | 203.0 |
| SC | 7.4-17.1 | 16.4 | 1718.0 | 1.22 (1.14-1.51) | 3.5 | 0.12 (0.10-0.32) | 200.9 |
| SP | 7.4-10.6 | 18.7 | 1375.7 | 1.03 (1.00-1.46) | 22.3 | 0.38 (0.10-0.64) | 2.9 |

T: Mean annual air temperature (in degrees Celsius, °C), P: Mean annual precipitation (in millimeters, mm), and BEF and R dif: the relative difference (in percentage, %) between the default values provided by MCTI (2004) and the values predicted by the models developed in this study, for BEF and R, respectively. These differences are calculated using Equation 13 from the paper.
